# Supplementary material for: Characterization and regulation of cell cycle-independent noncanonical gene targeting
Source: Nat Commun. 2024 Jun 18;15:5044. doi: 10.1038/s41467-024-49385-9 (PMC11189520; doi:10.1038/s41467-024-49385-9)
Supplement: Supplementary file 1 — Supplementary Information [file 41467_2024_49385_MOESM1_ESM.pdf]

Supplementary Information

Characterization and regulation of cell cycle-independent noncanonical gene targeting

Shinta Saito[1] and Noritaka Adachi[1,*]

1 Department of Life and Environmental System Science, Graduate School of Nanobioscience, Yokohama City University, Yokohama 236-0027, Japan

*Correspondence and requests for materials should be addressed to N.A. (nadachi@yokohama-cu.ac.jp)

Contents

- Supplementary Figures 1 – 13
- Supplementary Tables 1 – 5
- Supplementary Methods

Supplementary Figures

Supplementary Fig. 1 | Impact of DSB repair deficiency on gene targeting.

Supplementary Fig. 2 | Comprehensive analysis of chromosomal DSB repair using DR-GFP and SA-GFP reporter constructs.

Supplementary Fig. 3 | Genetic modification of the *MSH2* locus in human Nalm-6 and HT1080 cell lines.

Supplementary Fig. 4 | Impact of sequence divergence in vector arms on gene targeting.

Supplementary Fig. 5 | Opposing effects by Rad51/Rad52 inhibition on gene targeting.

Supplementary Fig. 6 | Genetic evidence for SSA-mediated integration events.

Supplementary Fig. 7 | Analysis of SSA-mediated DSB joining in *LIG4*$^{-/-}$*POLQ*$^{-/-}$ cells.

Supplementary Fig. 8 | Comprehensive analysis of SSA-mediated gene targeting.

Supplementary Fig. 9 | Analysis of cell-cycle dependence of gene targeting.

Supplementary Fig. 10 | Impact of BLM deficiency on gene targeting.

Supplementary Fig. 11 | Analysis of SSA-mediated gene targeting at exon 6 of the *HPRT* gene.

Supplementary Fig. 12 | SSA-dependent gene targeting in the HR-deficient cancer cell line MDA-MB-436.

Supplementary Fig. 13 | Comprehensive summary of HR- and SSA-based gene targeting.

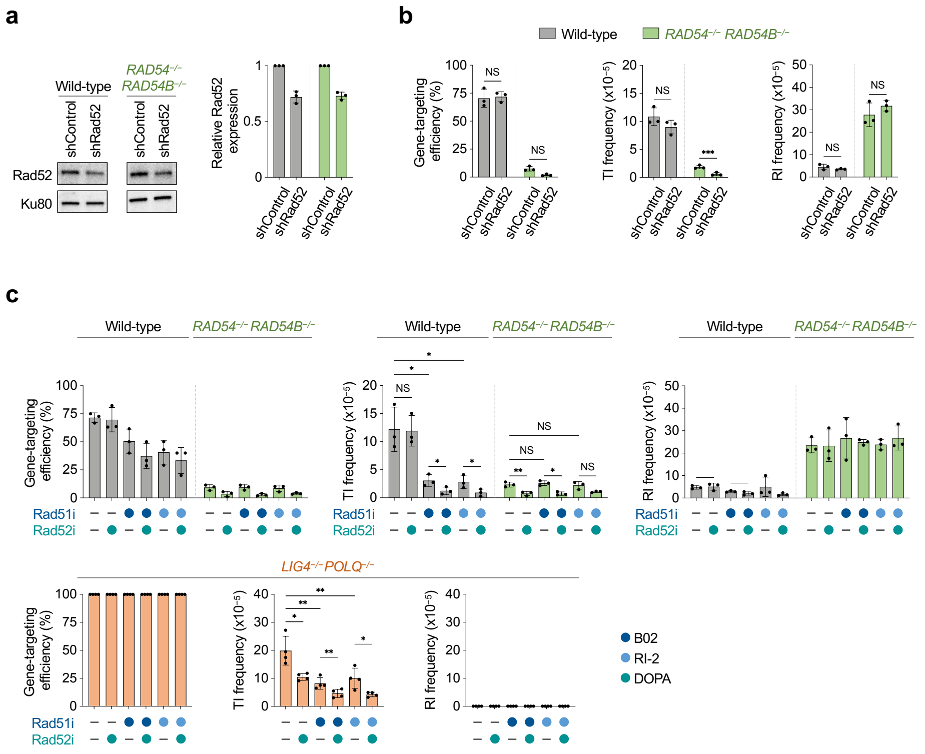

Supplementary Fig. 1 | Impact of DSB repair deficiency on gene targeting.
a, Western blot analysis for Rad52 in shRNA-transfected Nalm-6 wild-type and *RAD54−/− RAD54B−/−* cells. Cells were transfected with either pshRad52 or pshControl, cultured for 48 hr, and subjected to western blot analysis. Data shown are the mean ± s.d. (*n* = 3). **b,** Impact of Rad52 knockdown on TI frequency in wild-type and *RAD54−/− RAD54B−/−* cells. shRNA-treated cells were transfected with p8.9HPRT-2A-Puro, cultured for 24 hr, and plated into agarose medium. Data shown are the mean ± s.d. (*n* = 3). **c,** Impact of simultaneous inhibition of Rad51 and Rad52 on TI frequency in wild-type, *RAD54−/− RAD54B−/−*, and *LIG4−/− POLQ−/−* cells. Cells transfected with p8.9HPRT-2A-Puro cells were treated with either or both of 10 μM DOPA and Rad51 inhibitor (10 μM B02 or 75 μM RI-2) for 24 hr. The data of TI frequency are the same as that in Fig. 1e. Data shown are the mean ± s.d. (*n* = 3). Statistical significance in **b** and **c** was determined by two-sided Student's t-test. *P < 0.05; **P < 0.01; ***P < 0.001; NS, not significant. Source data are provided as a Source Data file.

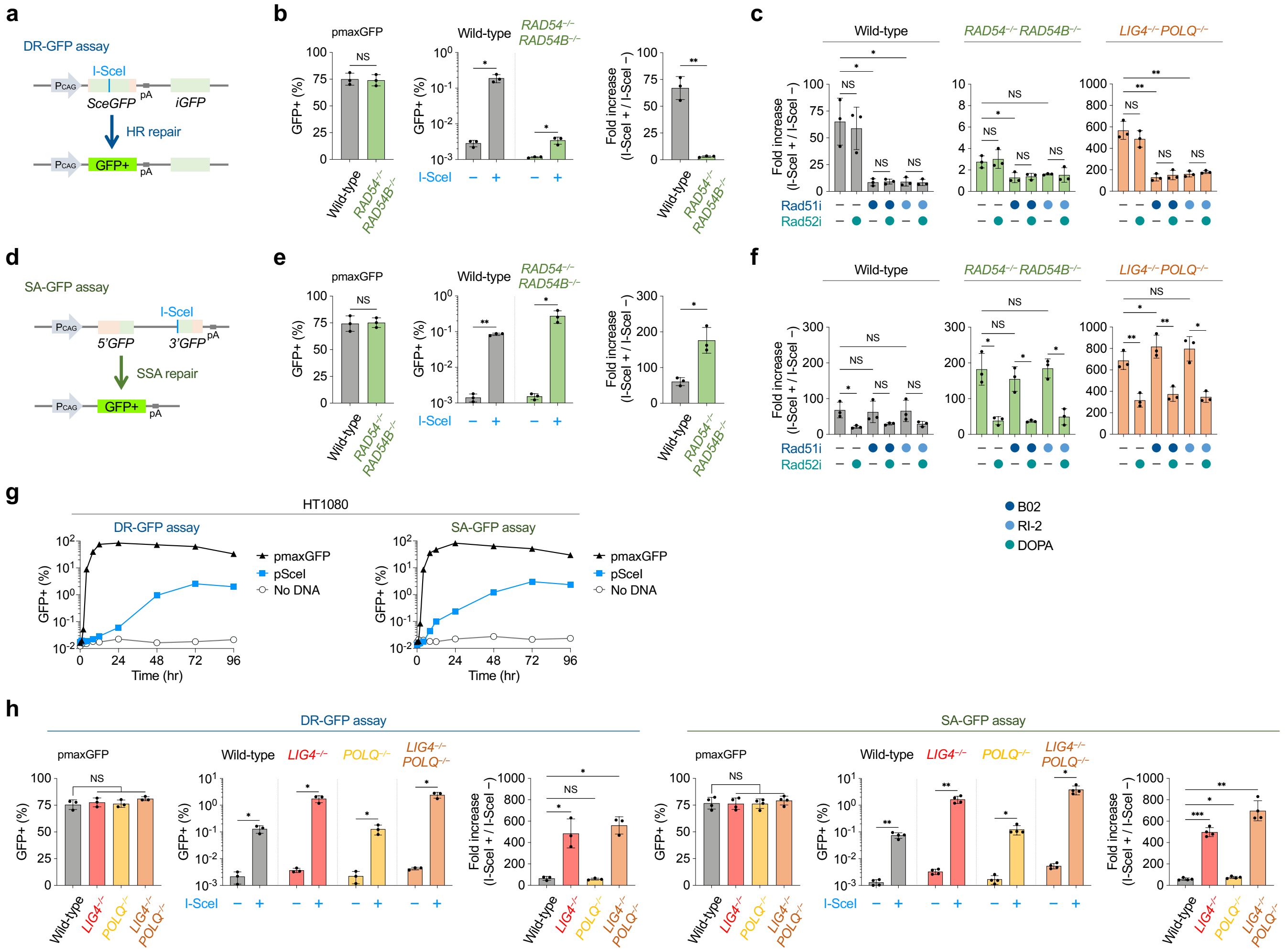

Supplementary Fig. 2 | Comprehensive analysis of chromosomal DSB repair using DR-GFP and SA-GFP reporter constructs.
a, Schematic of DR-GFP assay. In each cell line, the DR-GFP reporter cassette was knocked in at exon 3 of the *HPRT* locus, and successfully gene-targeted cells were used for experiments. GFP expression only occurs after recombination (HR repair) between *SceGFP* and *iGFP*. **b,** HR frequency in wild-type and *RAD54*$^{-/-}$ *RAD54B*$^{-/-}$ cells. Cells with the DR-GFP reporter construct were transfected with the I-SceI expression vector pSceI or the GFP expression vector pmaxGFP (which serves as a control for comparison of transfection efficiency). Cells were then cultured for 72 hr, and GFP-positive cells were counted. Data shown are the mean ± s.d. (n = 3). **c,** Impact of Rad51/Rad52 inhibition on HR frequency in wild-type, *RAD54*$^{-/-}$ *RAD54B*$^{-/-}$, and *LIG4*$^{-/-}$ *POLQ*$^{-/-}$ cells. After transfection of pSceI, cells were treated with either or both of 10 μM DOPA and Rad51 inhibitor (10 μM B02 or 75 μM RI-2) for 24 hr, and cultured for an additional 48 hr before counting GFP-positive cells. Data shown are the mean ± s.d. (n = 3). **d,** Schematic of SA-GFP assay. In each cell line, the SA-GFP reporter cassette was knocked in at exon 3 of the *HPRT* locus, and successfully gene-targeted cells were used for experiments. GFP expression only occurs after recombination (SSA repair) between *5'GFP* and *3'GFP*. **e,** SSA frequency in wild-type and *RAD54*$^{-/-}$ *RAD54B*$^{-/-}$ cells. Cells with the SA-GFP construct were transfected with pSceI or pmaxGFP. GFP-positive cells were counted after 72 hr of incubation. Data shown are the mean ± s.d. (n = 3). **f,** Impact of Rad51/Rad52 inhibition on SSA frequency in wild-type, *RAD54*$^{-/-}$ *RAD54B*$^{-/-}$, and *LIG4*$^{-/-}$ *POLQ*$^{-/-}$ cells. Experiments were performed as in **c**. Data shown are the mean ± s.d. (n = 3). **g,** Time-course analysis of HR and SSA frequencies in HT1080 cells. Cells with the DR-GFP or the SA-GFP reporter cassette were transfected with the I-SceI expression vector pSceI or the GFP expression vector pmaxGFP. Cells were then cultured for 1, 2, 4, 8, 12, 24, 48, 72, or 96 hr, and GFP-positive cells were counted at each time point. **h,** Impact of *LIG4* and/or *POLQ* knockout on HR- or SSA-mediated DSB repair. Cells with the DR-GFP or SA-GFP reporter cassette were transfected with pSceI or pmaxGFP. GFP-positive cells were counted after 72 hr of incubation. Data shown are the mean ± s.d. (DR-GFP assay, n = 3; SA-GFP assay, n = 4). Statistical significance in **b, c, e, f,** and **h** was determined by two-sided Student's t-test. *P < 0.05; **P < 0.01; ***P < 0.001; NS, not significant. Source data are provided as a Source Data file.

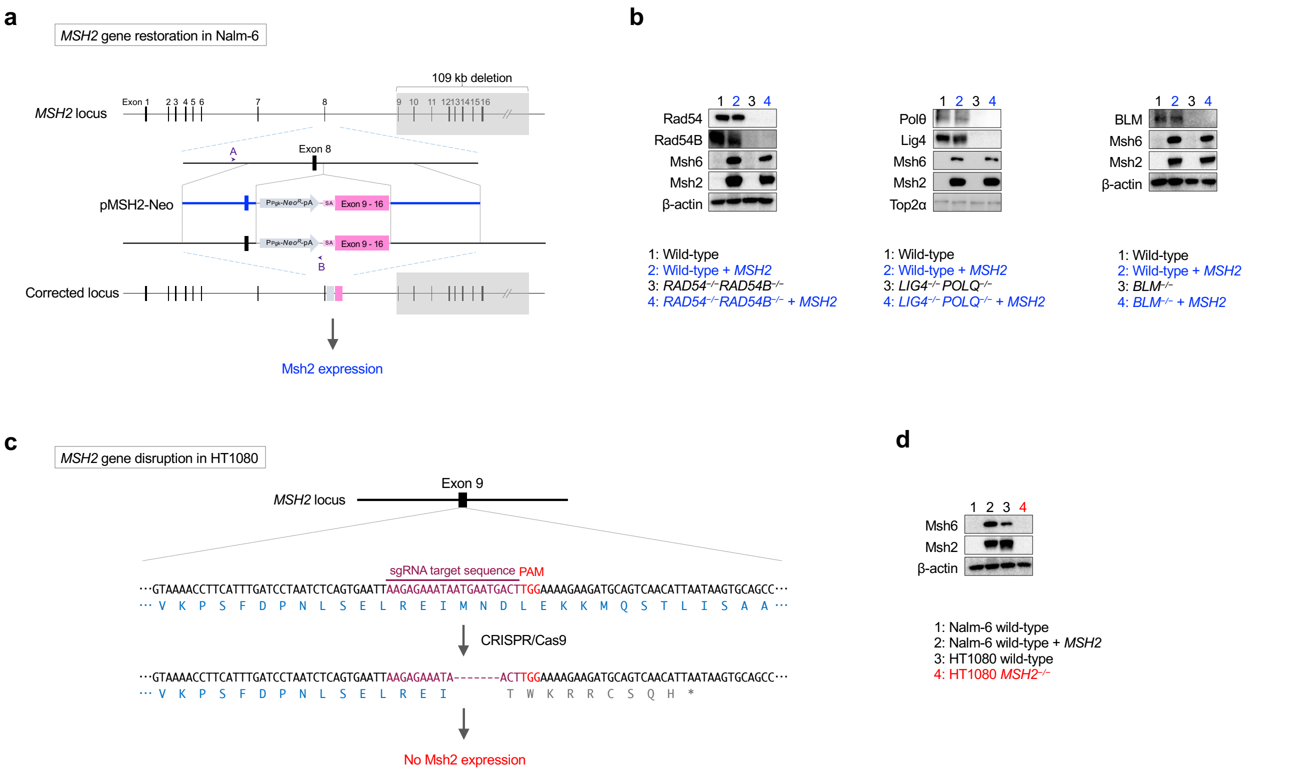

Supplementary Fig. 3 | Genetic modification of the *MSH2* locus in human Nalm-6 and HT1080 cell lines.
a, Scheme of restoration of Msh2 expression in Nalm-6 cells. When pMSH2-Neo is homologously integrated into the genome, a super-exon (exon 9 to 16) of *MSH2* is inserted, resulting in Msh2 expression from the endogenous promoter. Correct gene-targeting events in G418-resistant cells were confirmed by PCR analysis using primers A and B. **b,** Western blot analysis for Msh2 and Msh6 in *MSH2*-corrected Nalm-6 cell lines. **c,** CRISPR/Cas9-mediated *MSH2* gene disruption in HT1080 cells. Note that disrupted alleles harbored identical mutations (7-bp deletion at exon 9). **d,** Western blot analysis for Msh2 and Msh6 in *MSH2*-disrupted HT1080 cells. Source data are provided as a Source Data file.

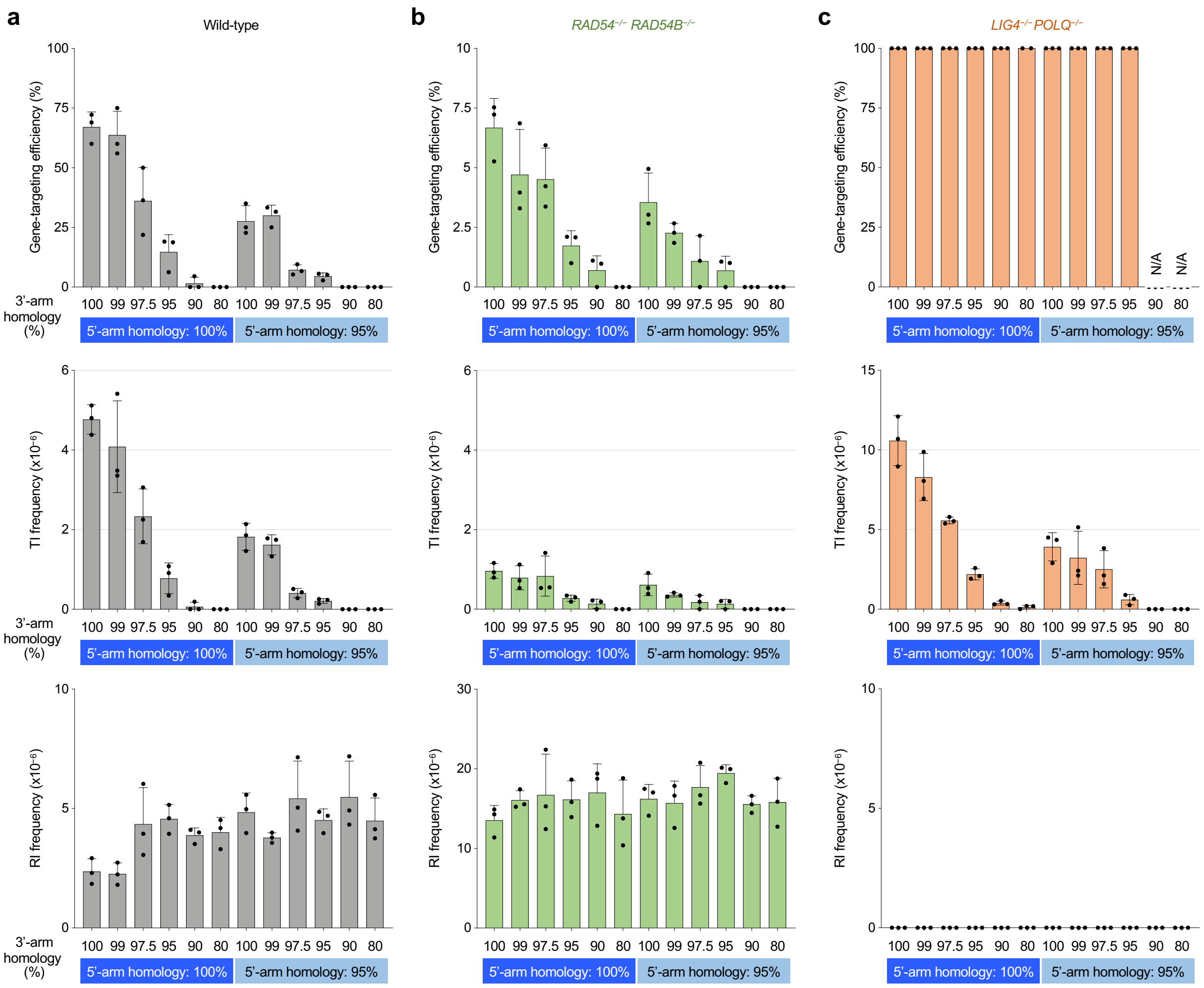

Supplementary Fig. 4 | Impact of sequence divergence in vector arms on gene targeting. Gene-targeting efficiency, TI frequency, and RI frequency of 12 *HPRT* targeting vectors shown in Fig. 2a in Nalm-6 wild-type (**a**), *RAD54*$^{-/-}$*RAD54B*$^{-/-}$ (**b**), and *LIG4*$^{-/-}$ *POLQ*$^{-/-}$ cells (**c**). Data shown are the mean ± s.d. (*n* = 3). The data of relative TI frequency in Fig. 2b are calculated from TI frequencies shown in this figure. N/A, not applicable. Source data are provided as a Source Data file.

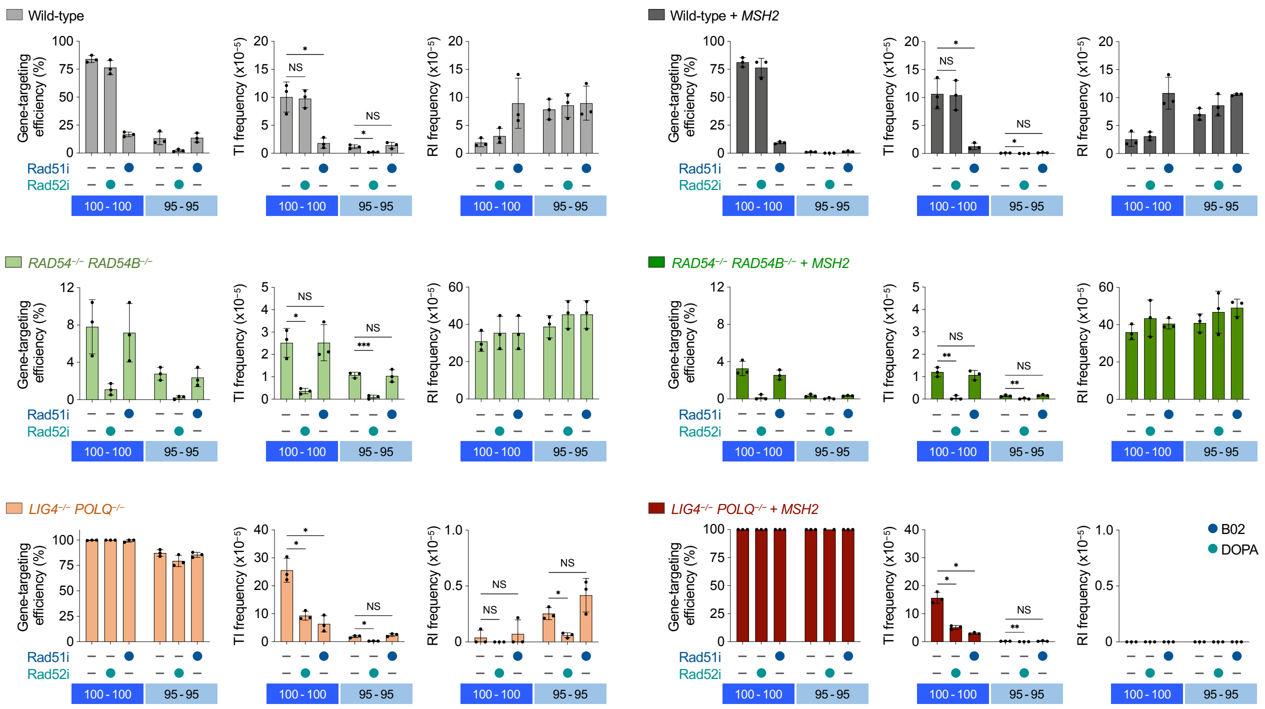

Supplementary Fig. 5 | Opposing effects by Rad51/Rad52 inhibition on gene targeting.
Gene-targeting efficiency, TI frequency, and RI frequency of p8.9HPRT-2A-Puro and p8.9HPRT-2A-Puro-95-95 vectors shown in Fig. 2e in Msh2-deficient (-) and proficient (+) cell lines from Nalm-6 wild-type, *RAD54*$^{-/-}$*RAD54B*$^{-/-}$, and *LIG4*$^{-/-}$ *POLQ*$^{-/-}$ cells. Data shown are the mean ± s.d. (*n* = 3). Note that the graphs for TI frequency in wild-type cell lines are the same as that in Fig. 2e and the graphs for *LIG4*$^{-/-}$ *POLQ*$^{-/-}$ cell lines are the same as that in Fig. 2f. Statistical significance was determined by two-sided Student's t-test. *P < 0.05; **P < 0.01; ***P < 0.001; NS, not significant. Source data are provided as a Source Data file.

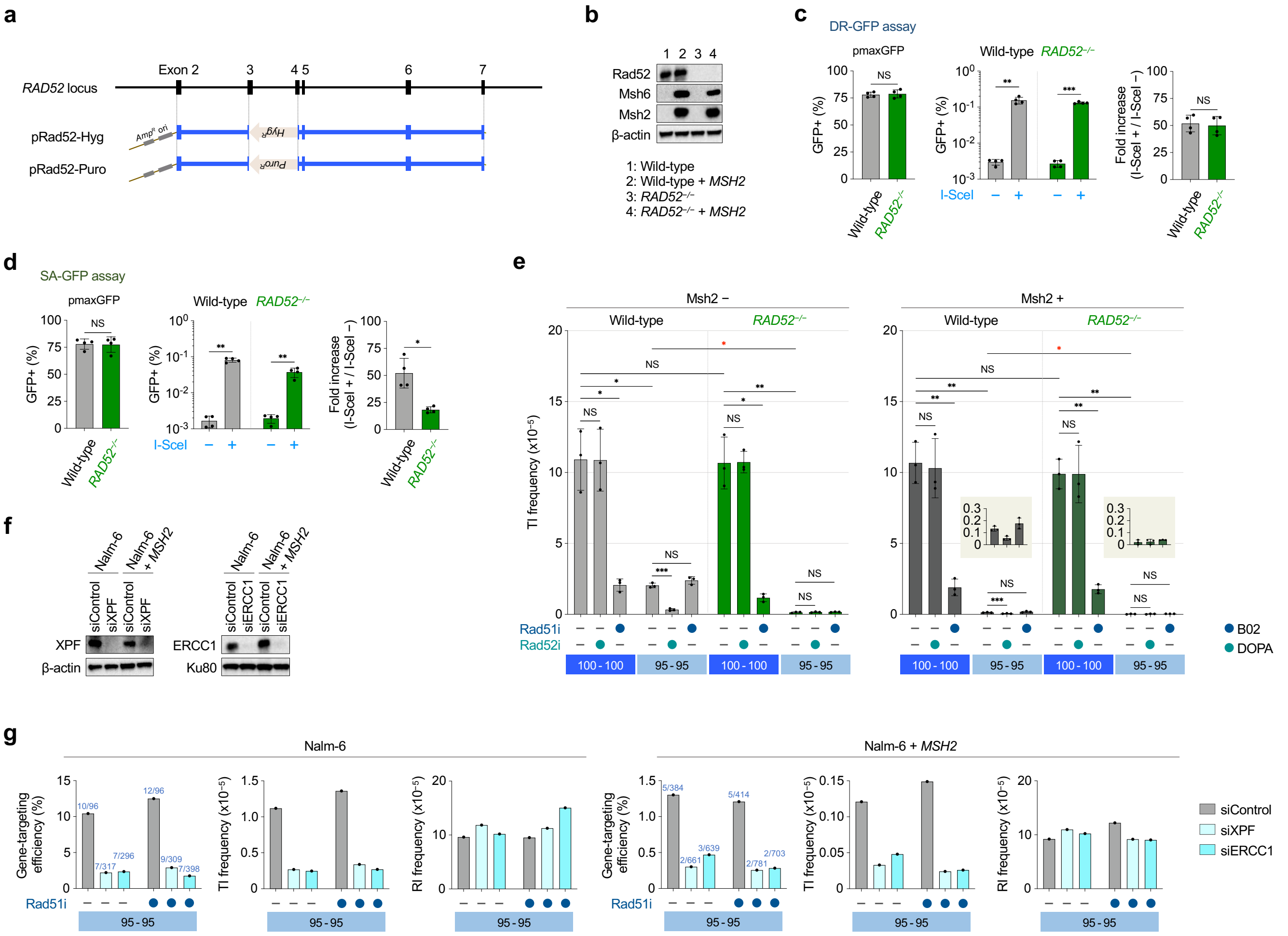

Supplementary Fig. 6 | Genetic evidence for SSA-mediated integration events.
a, Scheme for *RAD52* targeting. *RAD52*-targeting vectors were designed to replace exons 3 and 4 with a hygromycin-resistance (*Hyg*^{R}) or puromycin-resistance (*Puro*^{R}) gene. **b,** Western blot analysis for Rad52, Msh2, and Msh6 in Msh2-deficient (-) and proficient (+) cell lines from *RAD52* $^{-/-}$ Nalm-6 cells. **c,** HR frequency in wild-type and *RAD52* $^{-/-}$ cells. The DR-GFP assay was performed using a *RAD52* $^{-/-}$ cell line, in which DR-GFP reporter cassette was stably integrated into exon 3 of the *HPRT* gene. Data shown are the mean ± s.d. (n = 4). **d,** SSA frequency in wild-type and *RAD52* $^{-/-}$ cells. The SA-GFP assay was performed using a cell line, in which SA-GFP reporter cassette was stably integrated into exon 3 of the *HPRT* gene. Data shown are the mean ± s.d. (n = 4). **e,** Impact of Rad52 deficiency on TI frequency. Msh2-deficient (-) and proficient (+) cell lines from Nalm-6 wild-type and *RAD52* $^{-/-}$ cells were used for gene-targeting assay. Cells were transfected with p8.9HPRT-2A-Bsr or p8.9HPRT-2A-Bsr-95-95, and treated with 10 μM DOPA or 10 μM B02 for 24 hr prior to replating. Data shown are the mean ± s.d. (n = 3). Statistical significance in **c, d,** and **e** was determined by two-sided Student's t-test. *P < 0.05; **P < 0.01; ***P < 0.001; NS, not significant. Source data are provided as a Source Data file. **f,** Western blot analysis for XPF and ERCC1 in siRNA-transfected Msh2- and Msh2+ cell lines. Cells were transfected with either siXPF, siERCC1, or siControl, cultured for 24 hr, and subjected to western blot analysis. **g,** Impact of XPF/ERCC1 knockdown on gene targeting in Msh2- and Msh2+ Nalm-6 cells. siRNA-treated cells were transfected with either p8.9HPRT-2A-Puro or p8.9HPRT-2A-Puro-95-95, and treated with 10 μM B02 prior to replating. The numbers shown in the graphs for gene-targeting efficiency indicate "the number of targeted clones / the number of clones analyzed".

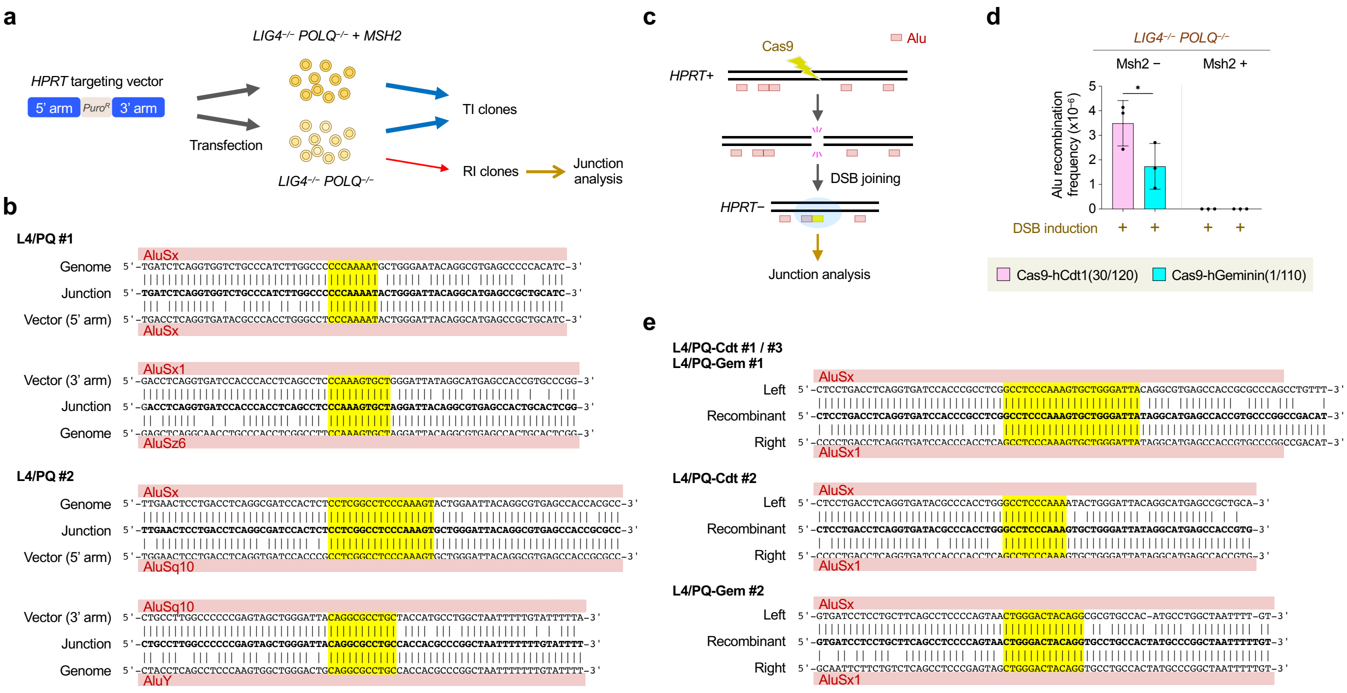

Supplementary Fig. 7 | Analysis of SSA-mediated DSB joining in *LIG4*$^{-/-}$*POLQ*$^{-/-}$ cells.

a, Analysis of random integrants (RI clones) from NHEJ/TMEJ-deficient Nalm-6 cells. The p8.9HPRT-2A-Puro vector was transfected into *LIG4*$^{-/-}$*POLQ*$^{-/-}$ or *LIG4*$^{-/-}$*POLQ*$^{-/-}$ + *MSH2* cells, and RI clones obtained from *LIG4*$^{-/-}$*POLQ*$^{-/-}$ cells were subjected to junction analysis. Note that no RI clones were obtained from *LIG4*$^{-/-}$*POLQ*$^{-/-}$ + *MSH2* cells (see Fig. 2f). **b,** Junction sequences of RI clones derived from *LIG4*$^{-/-}$*POLQ*$^{-/-}$ cells. Junctions analysed (L4/PQ #1 and #2) were found to utilize SSA-mediated recombination between Alu elements (AluSx, AluSx1, AluSz6, AluSq10, or AluY in this figure), one of which resides in the homology arm of the vector and the other in the genome. The SSA-mediated RI events rely on ≥ 8 bp homology, a tendency clearly distinguished from POLQ-dependent TMEJ that favors ≤ 6 bp of microhomology. **c,** Scheme of chromosomal DSB joining assay. After DSB induction at the *HPRT* locus (exon 3) by Cas9-hCdt1(30/120) or Cas9-hGeminin(1/110) vector transfection, 6TG-resistant (*HPRT*-deficient) clones were subjected to junction analysis. **d,** Alu recombination frequency in *LIG4*$^{-/-}$*POLQ*$^{-/-}$ (Msh2-) and *LIG4*$^{-/-}$*POLQ*$^{-/-}$ + *MSH2* (Msh2+) cells. Data shown are the mean ± s.d. (n = 3). Statistical significance was determined by two-sided Student's t-test. *P < 0.05. Source data are provided as a Source Data file. **e,** Junction sequences of Alu recombinants from *LIG4*$^{-/-}$*POLQ*$^{-/-}$ cells. L4/PQ-Cdt #1 - #3 are derived from Cas9-hCdt1(30/120)-transfected cells, while L4/PQ-Gem #1 and #2 are derived from Cas9-hGeminin(1/110)-transfected cells. All junctions analysed were dependent on SSA recombination between two separate Alu elements, similar to that seen in rare RI clones shown in **b**. Note that Alu recombinants were not obtained from *LIG4*$^{-/-}$*POLQ*$^{-/-}$ + *MSH2* cells.

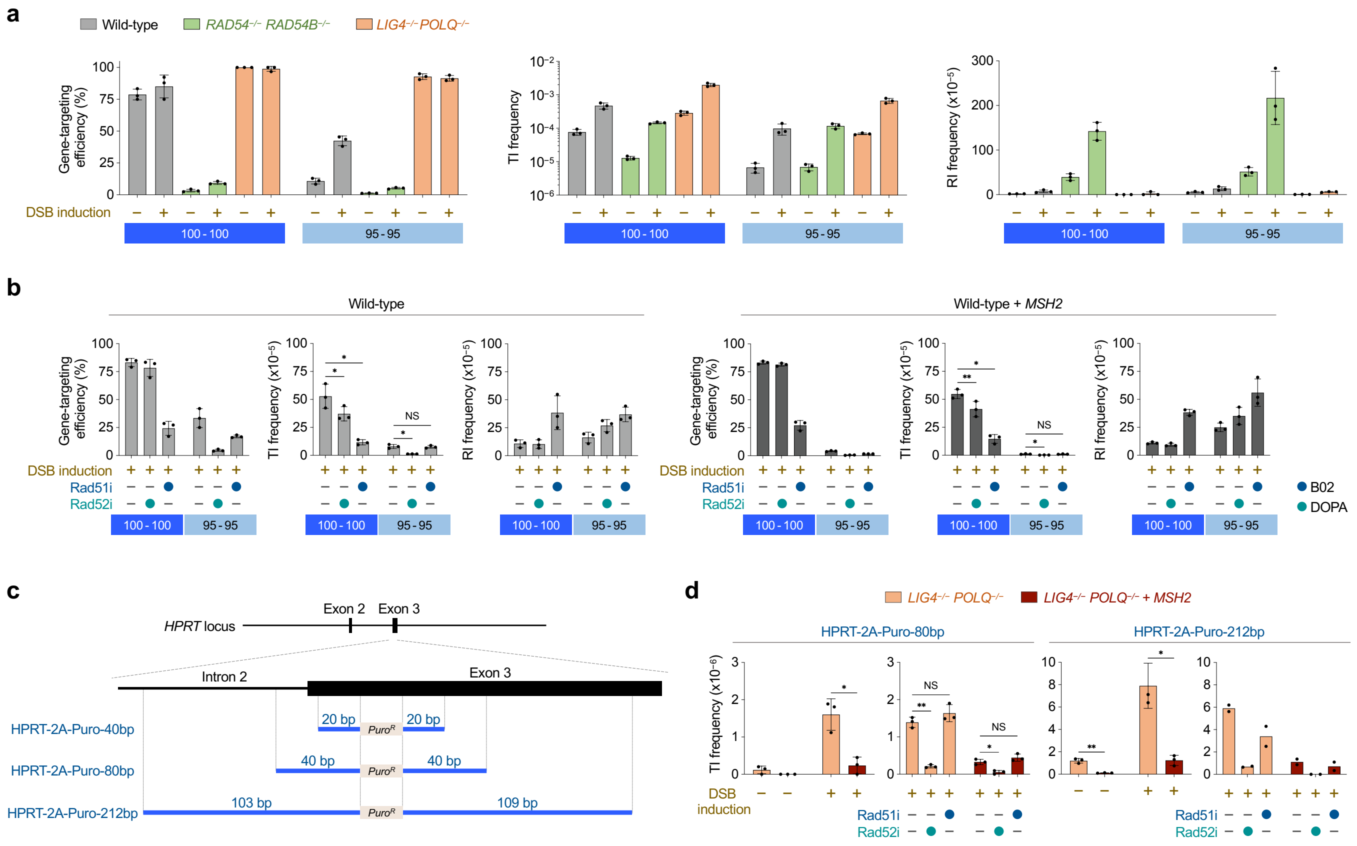

Supplementary Fig. 8 | Comprehensive analysis of SSA-mediated gene targeting. **a,** Impact of Cas9-induced DNA cleavage on gene targeting. Nalm-6 wild-type, *RAD54*$^{-/-}$ *RAD54B*$^{-/-}$, and *LIG4*$^{-/-}$ *POLQ*$^{-/-}$ cells were transfected with p8.9HPRT-2A-Puro (100-100) or p8.9HPRT-2A-Puro-95-95 (95-95) along with pX330-Cas9-HPRT-Ex3 (for DSB induction) or pX330-U6-Chimeric_BB-CBh-hSpCas9 (negative control). Data shown are the mean ± s.d. (n = 3). The data of Fig. 3b are calculated from TI frequencies shown in this figure. **b,** Impact of Rad51/Rad52 inhibition on Cas9 DSB-induced gene targeting. Nalm-6 Msh2- and Msh2+ cell lines were subjected to co-transfection of targeting vector and pX330-Cas9-HPRT-Ex3 as in **a**, and treated with 10 μM DOPA or 10 μM B02 for 24 hr. Data shown are the mean ± s.d. (n = 3). The data of relative TI frequency in Fig. 3c are calculated from TI frequencies shown in this figure. Statistical significance was determined by Student's t-test. *P < 0.05; **P < 0.01; NS, not significant. **c,** Schematic representation of *HPRT* targeting vectors with short homology arms. Shown are 3 targeting vectors with a total arm length of 40 bp, 80 bp, or 212 bp. **d,** Gene-targeting assay using the short-arm *HPRT* targeting vectors. Msh2- and Msh2+ *LIG4*$^{-/-}$ *POLQ*$^{-/-}$ cells were transfected with HPRT-2A-Puro-40bp, HPRT-2A-Puro-80bp, or HPRT-2A-Puro-212bp along with pX330-Cas9-HPRT-Ex3 (for DSB induction) or pX330-U6-Chimeric_BB-CBh-hSpCas9 (negative control), and treated with 10 μM DOPA or 10 μM B02 for 24 hr prior to replating. Symbols are as in **b**. Data shown are the mean ± s.d. (n = 3). Note that HPRT-2A-Puro-40bp was incompetent at gene targeting. Statistical significance in **b** and **d** was determined by two-sided Student's t-test. *P < 0.05; **P < 0.01; NS, not significant. Source data are provided as a Source Data file.

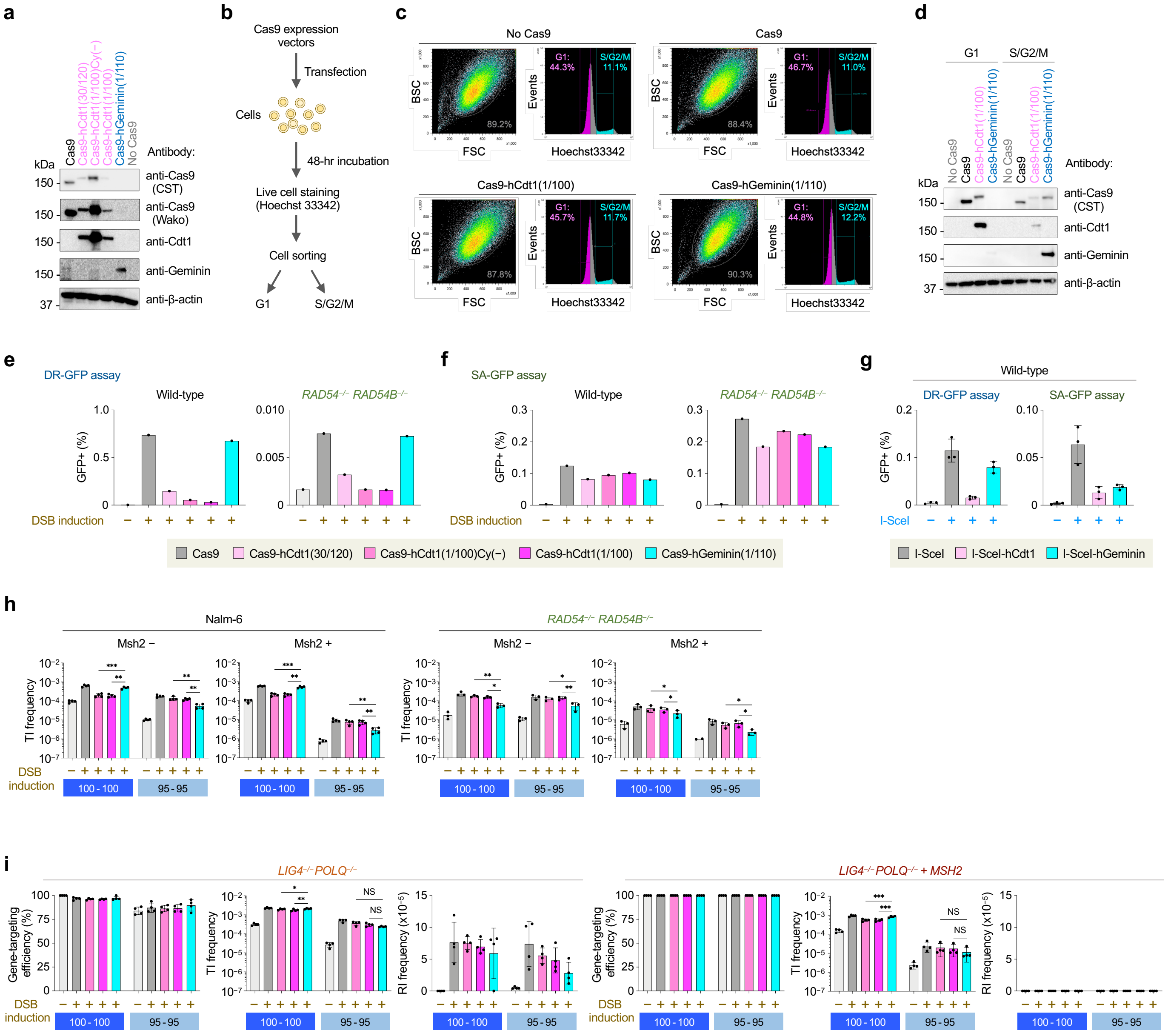

Supplementary Fig. 9 | Analysis of cell-cycle dependence of gene targeting.

a, Western blot analysis for cell cycle-regulated Cas9 proteins. HT1080 cells were transfected with either pX330-Cas9-HPRT-Ex3, pX330-Cas9-hCdt1(30/120)-Ex3, pX330-Cas9-hCdt1(1/100)Cy(-)-Ex3, pX330-Cas9-hCdt1(1/100)-HPRT-Ex3, or pX330-Cas9-hGeminin(1/110)-HPRT-Ex3, cultured for 48 hr, and subjected to western blot analysis. CST, Cell Signaling Technology; WAKO, FUJIFILM Wako Pure Chemical. **b,** Scheme of cell sorting-based analysis of Cas9 expression. HT1080 cells were transfected with either pX330-Cas9-HPRT-Ex3, pX330-Cas9-hCdt1(1/100)-HPRT-Ex3, or pX330-Cas9-hGeminin(1/110)-HPRT-Ex3, and cultured for 48 hr. Cells were then stained with 5 μg/ml Hoechst 33342 and sorted according to their DNA content. After sorting, cells were subjected to western blot analysis. **c,** Gate settings for cell sorting. Cells were gated according to forward scatter (FSC) and backscatter (BSC) parameters. Isolated G1 cells correspond to the area shown in magenta (note that contamination of early S-phase cells is thus avoided), while S/G2/M cells correspond to the area shown in light blue. **d,** Western blot analysis for cell cycle-regulated Cas9 proteins from G1 and S/G2/M cells. **e,** Impact of cell cycle-regulated Cas9 expression on chromosomal HR repair in Nalm-6 wild-type and *RAD54*$^{-/-}$ *RAD54B*$^{-/-}$ cells. **f,** Impact of cell cycle-regulated Cas9 expression on chromosomal SSA repair in Nalm-6 wild-type and *RAD54*$^{-/-}$ *RAD54B*$^{-/-}$ cells. **g,** Impact of cell cycle-regulated I-SceI expression on chromosomal DSB repair via HR and SSA. **h,** Impact of targeted DSB induction on TI frequency in Msh2- and Msh2+ Nalm-6 wild-type and *RAD54*$^{-/-}$ *RAD54B*$^{-/-}$ cells. Cells were transfected with p8.9HPRT-2A-Puro (100-100) or p8.9HPRT-2A-Puro-95-95 (95-95) with or without Cas9 expression vector. The data shown in Fig. 3g are calculated from TI frequencies shown in this figure. Data shown are the mean ± s.d. (n = 3). **i,** Impact of targeted DSB induction on gene targeting in Msh2- and Msh2+ *LIG4*$^{-/-}$ *POLQ*$^{-/-}$ cells. Cells were transfected with p8.9HPRT-2A-Puro (100-100) or p8.9HPRT-2A-Puro-95-95 (95-95) with or without Cas9 expression vector. The data shown in Fig. 3g are calculated from TI frequencies shown in this figure. Data shown are the mean ± s.d. (n = 3). Statistical significance in **h** and **i** was determined by two-sided Student's t-test. *P < 0.05; **P < 0.01; ***P < 0.001; NS, not significant. Source data are provided as a Source Data file.

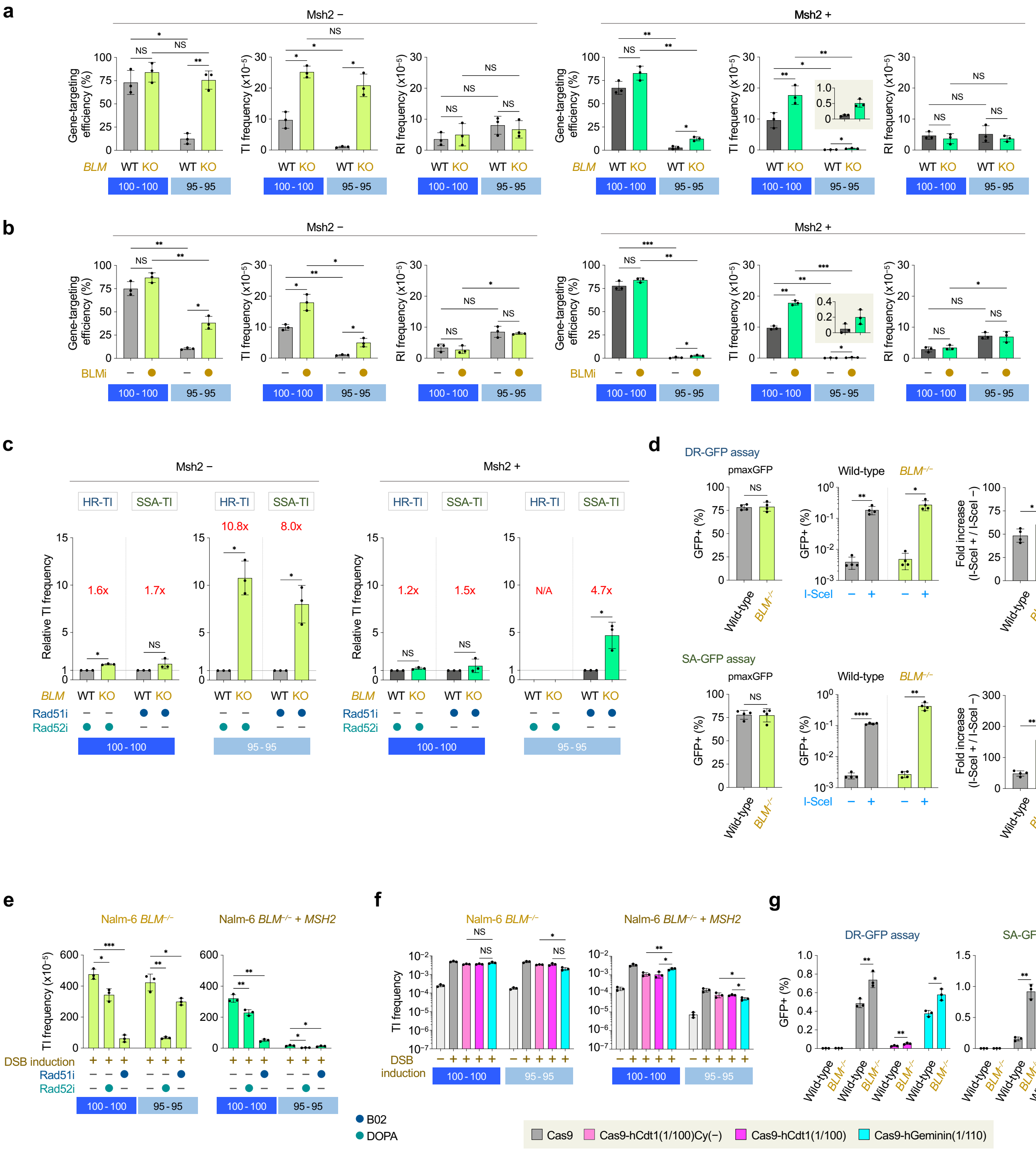

Supplementary Fig. 10 | Impact of BLM deficiency on gene targeting.
a, Impact of *BLM* gene-knockout on gene targeting. Nalm-6 Msh2- and Msh2+ cells proficient (WT) or deficient (KO) in *BLM* were transfected with p8.9HPRT-2A-Puro (100-100) or p8.9HPRT-2A-Puro-95-95 (95-95). Data shown are the mean ± s.d. (*n* = 3), and the data of TI frequency of 100-100 and 95-95 vectors are the same as that in Fig. 4a. **b,** Impact of BLM inhibition on gene targeting. Nalm-6 Msh2+ and Msh2- cells were transfected with p8.9HPRT-2A-Puro (100-100) or p8.9HPRT-2A-Puro-95-95 (95-95), and treated with 5 μM ML216 for 24 hr prior to replating. Data shown are the mean ± s.d. (*n* = 3). **c,** Impact of BLM deficiency on HR-TI and SSA-TI. Nalm-6 Msh2- and Msh2+ cells proficient (WT) or deficient (KO) in *BLM* were transfected with p8.9HPRT-2A-Puro (100-100) or p8.9HPRT-2A-Puro-95-95 (95-95). The TI frequency of p8.9HPRT-2A-Puro (100-100) or p8.9HPRT-2A-Puro-95-95 (95-95) in Msh2- and Msh2+ cells proficient (WT) in *BLM* was taken as 1, and the relative TI frequency was calculated. Data shown are the mean ± s.d. (*n* = 3), and the data of TI frequency of 100-100 and 95-95 vectors of *BLM* KO cells are the same as that in Fig. 4c. N/A, not applicable. **d,** HR and SSA frequency in wild-type and *BLM* $^{-/-}$ cells. Cells with the DR-GFP or SA-GFP reporter construct were transfected with the I-SceI expression vector pSceI or the GFP expression vector pmaxGFP. Cells were then cultured for 72 hr, and GFP-positive cells were counted. Data shown are the mean ± s.d. (*n* = 4). **e,** Impact of Rad51/Rad52 inhibition on the frequency of Cas9 DSB-induced TI in *BLM* $^{-/-}$ cells. Cells were electroporated with p8.9HPRT-2A-Puro (100-100) or p8.9HPRT-2A-Puro-95-95 (95-95) with or without Cas9 expression vector. Data shown are the mean ± s.d. (*n* = 3). **f,** Impact of targeted DSB induction on gene targeting in *BLM* $^{-/-}$ cells. Cells were transfected with p8.9HPRT-2A-Puro (100-100) or p8.9HPRT-2A-Puro-95-95 (95-95) with or without Cas9 expression vector. The data shown in Fig. 4d are calculated from the TI frequency shown in this figure. Data shown are the mean ± s.d. (*n* = 3). **g,** Impact of cell cycle-regulated Cas9 expression on chromosomal HR and SSA repair in *BLM* $^{-/-}$ cells. Scheme for DR-GFP and SA-GFP assays are shown in Fig. 3e,f. The cells were transfected with each Cas9 expression vector, and GFP-positive cells were counted. Data shown are the mean ± s.d. (*n* = 3). Statistical significance in **a-g** was determined by two-sided Student's t-test. *P < 0.05; **P < 0.01; ***P < 0.001; ****P < 0.0001; NS, not significant. Source data are provided as a Source Data file.

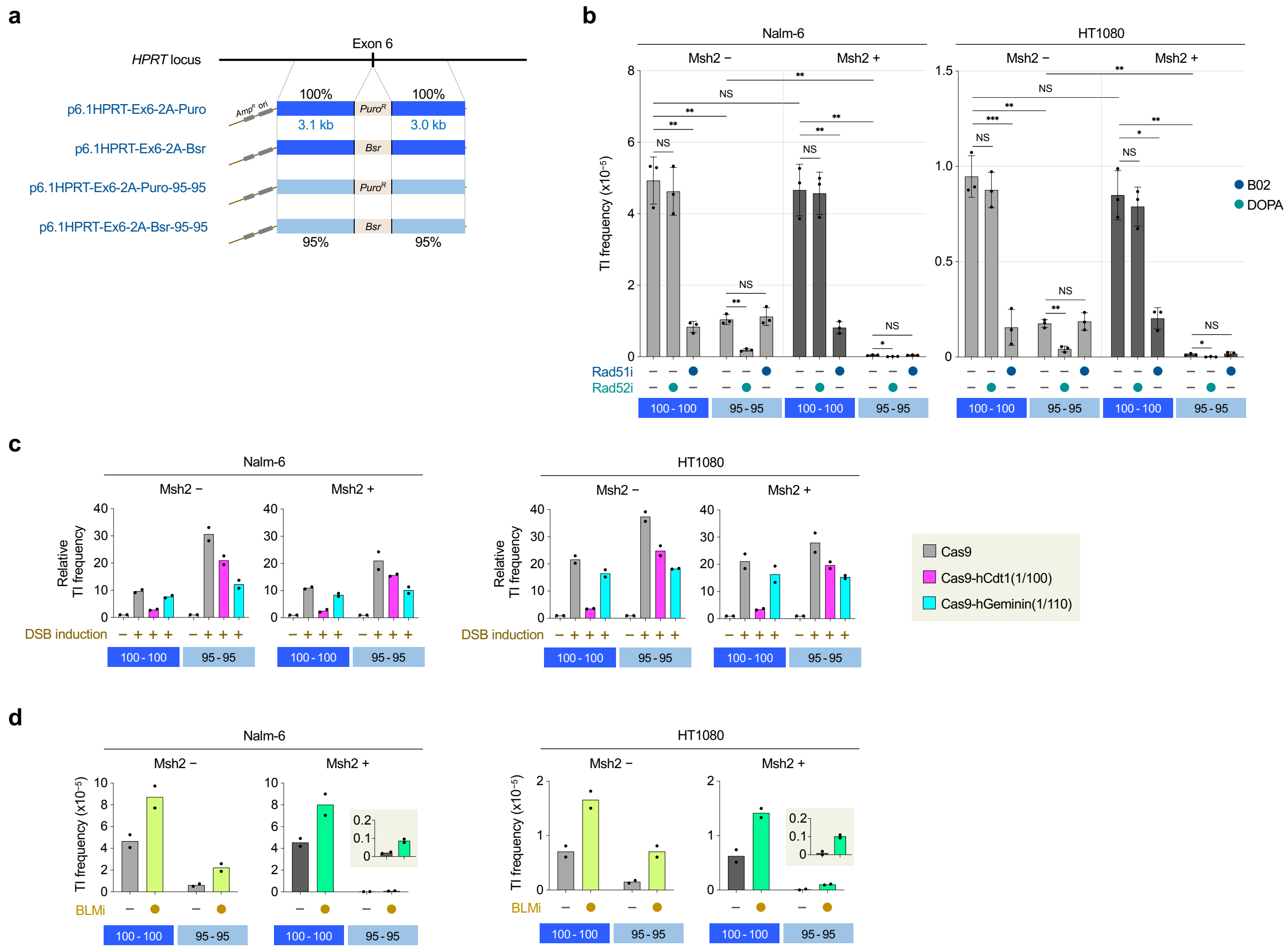

Supplementary Fig. 11 | Analysis of SSA-mediated gene targeting at exon 6 of the *HPRT* gene.
a, Schematic representation of targeting vectors for disruption of exon 6 of the *HPRT* gene. Targeting vectors were designed to insert a puromycin-resistance (*Puro*^{R}) or blasticidin-resistance (*Bsr*) gene cassette into exon 6 of the human *HPRT* gene. Unlike p6.1HPRT-Ex6-2A-Puro and p6.1HPRT-Ex6-2A-Bsr, the p6.1HPRT-Ex6-2A-Puro-95-95 and p6.1HPRT-Ex6-Bsr-95-95 vectors harbor 5% sequence divergence (one mismatch every 20 bp) in both arms. **b,** Impact of Msh2 deficiency on TI frequency. Msh2-deficient (-) and proficient (+) cell lines from Nalm-6 and HT1080 were used for gene-targeting assay. For Nalm-6, cells were transfected with p6.1HPRT-Ex6-2A-Puro or p6.1HPRT-Ex6-2A-Puro-95-95, and treated with 10 μM DOPA or 10 μM B02 for 24 hr; for HT1080, cells were transfected with p6.1HPRT-Ex6-2A-Bsr or p6.1HPRT-Ex6-2A-Bsr-95-95, and treated with 10 μM DOPA or 10 μM B02 for 48 hr prior to replating. Data shown are the mean ± s.d. (n = 3). Statistical significance was determined by two-sided Student's t-test. *P < 0.05; **P < 0.01; ***P < 0.001; NS, not significant. Source data are provided as a Source Data file. **c,** Impact of targeted DSB induction on TI frequency. Msh2-deficient (-) and proficient (+) cell lines from Nalm-6 and HT1080 were used for gene-targeting assay. For Nalm-6, cells were transfected with p6.1HPRT-Ex6-2A-Puro (100-100) or p6.1HPRT-Ex6-2A-Puro-95-95 (95-95) with or without Cas9 expression vector. For HT1080, cells were transfected with p6.1HPRT-Ex6-2A-Bsr (100-100) or p6.1HPRT-Ex6-2A-Bsr-95-95 (95-95) with or without Cas9 expression vector. The TI frequency of the 100-100 or 95-95 vectors without Cas9 in each cell line was taken as 1, and the relative TI frequency was calculated. Data shown are the mean of two independent experiments. **d**, Impact of BLM inhibition on TI frequency. Msh2-deficient (-) and proficient (+) cell lines from Nalm-6 and HT1080 were used for gene-targeting assay. For Nalm-6, cells were transfected with p6.1HPRT-Ex6-2A-Puro (100-100) or p6.1HPRT-Ex6-2A-Puro-95-95 (95-95), and treated with 5 μM ML216 for 24 hr; for HT1080, cells were transfected with p6.1HPRT-Ex6-2A-Bsr (100-100) or p6.1HPRT-Ex6-2A-Bsr-95-95 (95-95), and treated with 5 μM ML216 for 24 hr prior to replating. Data shown are the mean of two independent experiments.

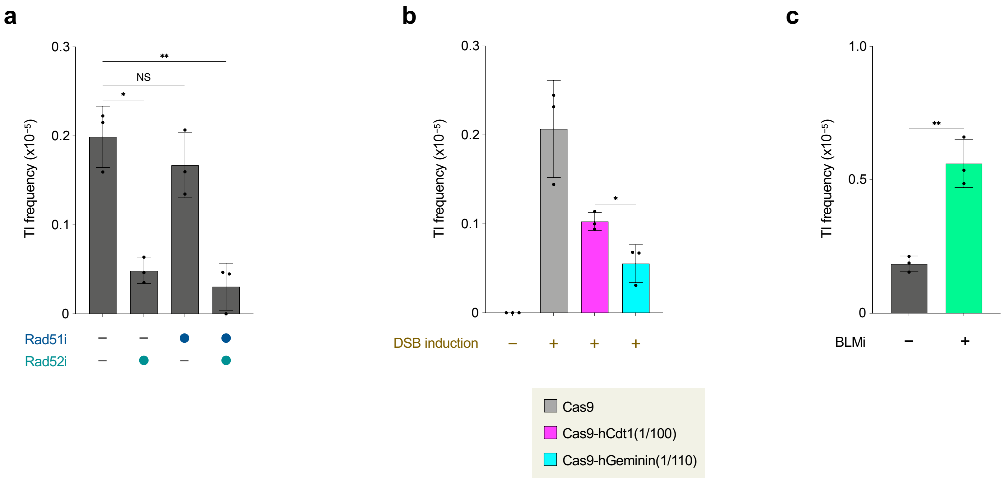

Supplementary Fig. 12 | SSA-dependent gene targeting in the HR-deficient cancer cell line MDA-MB-436.
a, Impact of inhibition of Rad51 and Rad52 on TI frequency. MDA-MB-436 cells were co-transfected with p8.9HPRT-2A-Puro and pX330-Cas9-HPRT-Ex3, and treated with either or both of 10 μM DOPA and 10 μM B02 for 48 hr prior to replating. Data shown are the mean ± s.d. (n = 3). **b,** Impact of cell cycle-regulated DSB induction on TI frequency. Cells were transfected with p8.9HPRT-2A-Puro with or without pX330-Cas9-HPRT-Ex3. Data shown are the mean ± s.d. (n = 3). **c**, Impact of BLM inhibition on TI frequency. Cells were transfected with p8.9HPRT-2A-Puro along with pX330-Cas9-HPRT-Ex3, and treated with BLM inhibitor (5 μM ML216) for 48 hr prior to replating. Statistical significance in **a-c** was determined by two-sided Student's t-test. *P < 0.05; **P < 0.01; NS, not significant. Source data are provided as a Source Data file.

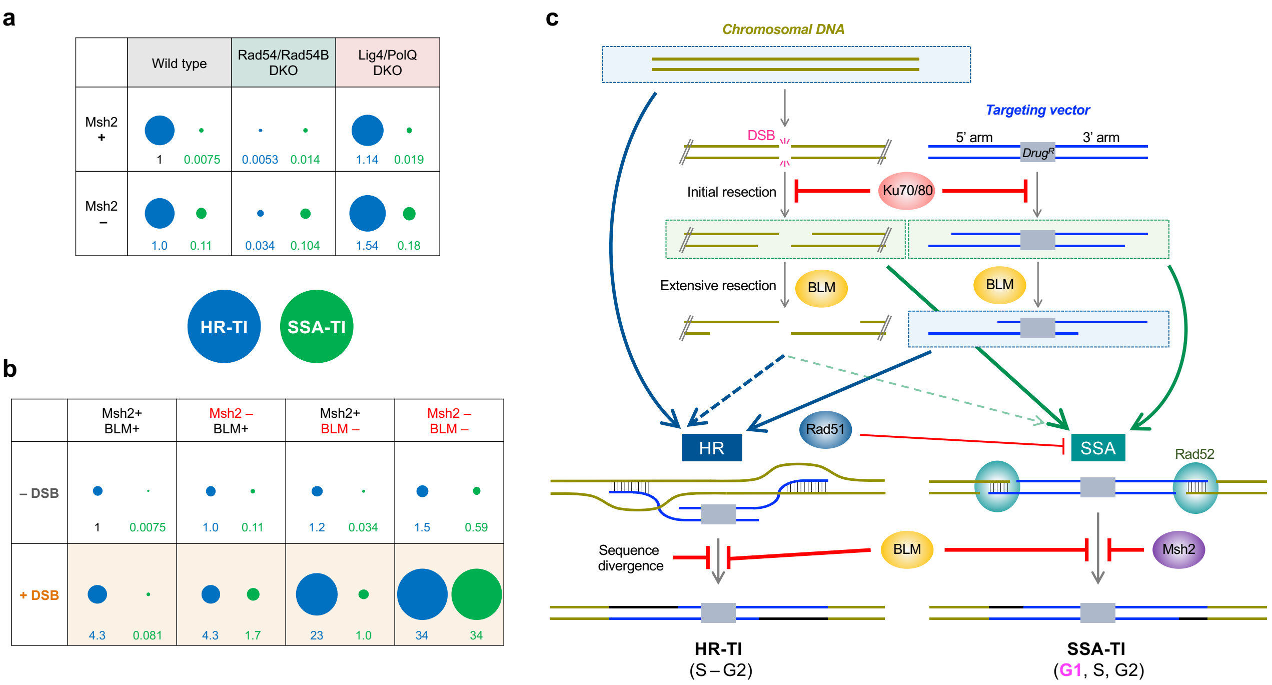

Supplementary Fig. 13 | Comprehensive summary of HR- and SSA-based gene targeting.
a, Relative frequencies of HR-TI and SSA-TI. The impacts of Msh2 status and of HR or NHEJ/TMEJ deficiency are indicated. Cell lines shown are: wild-type, Nalm-6; Rad54/Rad54B DKO (double-knockout), *RAD54*$^{-/-}$ *RAD54B*$^{-/-}$; and Lig4/PolQ DKO, *LIG4*$^{-/-}$ *POLQ*$^{-/-}$. The data are based on the results shown in Fig. 2e,f and Supplementary Fig. 5. Note that the mean HR-TI frequency in Msh2+ Nalm-6 is taken as 1. **b,** Relative frequencies of HR-TI and SSA-TI after Cas9-mediated targeted genome DNA cleavage. The impacts of DSB induction and of Msh2 and/or BLM deficiency are indicated. The data is based on the results shown in Figs. 2e, 3c,g, and 4c,d and Supplementary Fig. 10e. Note that the mean HR-TI frequency in Msh2+ Nalm-6 (i.e., Msh2+/BLM+ cells without DSB induction) is taken as 1. **c,** Model for HR-TI and SSA-TI. This study has revealed the existence of SSA-TI, which is promoted by Rad52 and does not rely on the HR protein Rad51. Unlike HR-TI, SSA-TI is negatively affected by Msh2 protein, is tolerant to sequence divergence, and is suggested to favor short-range resected DSB ends (after initial resection), which may preferentially be produced in the absence of BLM, as this protein promotes extensive resection to form long-range resected DSB ends, substrates required to initiate HR.

Supplementary Tables

Supplementary Table 1. Oligonucleotides used in this study.

Supplementary Table 2. Cas9/sgRNA expression vectors used in this study.

Supplementary Table 3. *HPRT* targeting vectors used in this study.

Supplementary Table 4. PCR primers used to amplify the homology arms of *HPRT* targeting vectors.

Supplementary Table 5. Antibodies used in this study.

Supplementary Table 1. Oligonucleotides used in this study.

Name	Sequence	Reference
HPRT-2A-Bsr-Fw	5'-GGACTGAACGTCTTGCTCGAGGGGAGGGCAGAGGAAGTCTTC-3'	This study
HPRT-2A-Bsr-Rv	5'-AGTGAGCGAGGAAGCTCGAGACAACATATCCAGTCACTATGG-3'	This study
HPRT-TV-backbone-Fw	5'-CAATGTATCTTAAGGATCCGCTGCATTAATGAATCGG-3'	This study
HPRT-TV-backbone-Rv	5'-CATGGTGGCTTAAGGATCCTTTGCCAAAATGATGAGACAGC-3'	This study
HPRT-20bp-Fw	5'-AACGTCTTGCTCGAGATGTGCAAG-3'	This study
HPRT-20bp-Rv	5'-ACAGAGGGCTACAATGTGATTCCAC-3'	This study
HPRT-40bp-Fw	5'-ATTTTATTTCTGTAGGACTGAACG-3'	This study
HPRT-40bp-Rv	5'-TTATAGCCCCCCTTGAGCACACAG-3'	This study
HPRT-100bp-Fw	5'-GTTTAATGACTAAGAGGTGTTTG-3'	This study
HPRT-100bp-Rv	5'-GTCATAGGAATGGATCTATCAC-3'	This study
Rad52-5-Fw	5'-TAACAGGGTAATGATCAGAATCAAGATGTCTGGGACTGAG-3'	This study
Rad52-5-Rv	5'-GGCCGCAGCCTGCTTCTGGTACTCTTCTGCTGTGTACTGG-3'	This study
Rad52-3-Fw	5'-AGGTCACTATCAGTCGATATCACTCCATCACGCAGCAGAATGTGG-3'	This study
Rad52-3-Rv	5'-TGCTCGACGCGTGATGTCGACGTGATCTCAGGTAGTCTTTGTCCAG-3'	This study
shRad52-top	5'-GATCCGGAAATATGATCCATCTTACTGTGAAGCCACAGATGGGTAAGATGGATCATATTTCCTTTTTTA-3'	This study
shRad52-btm	5'-AGCTTAAAAAAGGAAATATGATCCATCTTACCCATCTGTGGCTTCACAGTAAGATGGATCATATTTCCG-3'	This study
shCtrl-top	5'-GATCCGCCTAAGGTTAAGTCGCCCTCTGTGAAGCCACAGATGGGAGGGCGACTTAACCTTAGGCTTTTTTA-3'	This study
shCtrl-btm	5'-AGCTTAAAAAAGCCTAAGGTTAAGTCGCCCTCCCATCTGTGGCTTCACAGAGGGCGACTTAACCTTAGGCG-3'	This study
Cas9-linker-top	5'-CCAGGCAAAAAAGAAAAAGAGCGCTGGATCCGCTAGCTAAG-3'	This study
Cas9-linker-btm	5'-AATTCTTAGCTAGCGGATCCAGCGCTCTTTTTCTTTTTTGCCTGGCCGG-3'	This study
Cas9-Geminin-Fw	5'-AAAAAGAGCGCTGGATCCGAGATGAATCCCAGTATGAAGCAG-3'	This study
Cas9-Geminin-Rv	5'-TTCTTAGCTAGCGGATCTCAGCGCCTTTCTCCGTTTTTCTGC-3'	This study
Cas9-Cdt1(30/120)-Fw	5'-AAAAAGAGCGCTGGATCCTTCGGATATCCATCACACTGGC-3'	This study
Cas9-Cdt1(30/120)-Rv	5'-TTCTTAGCTAGCGGATCTGATGGTGTCCTGGTCCTGCGCG-3'	This study
Cas9-Cdt1(1/100)-Fw	5'-CACACTGGCGGCCGCTCGAGATGGAGCAGCGCCGCGTCACCGAC-3'	This study
Cas9-Cdt1(1/100)-Rv	5'-TCAGCGAGCTCTAGGAATTCTTATTTCTTTATCTTCTGGCCCGGAG-3'	This study
Cas9-Cdt1(1/100)Cy(-)-Fw	5'-GCCCGCGCGGCCGCGCGGCTGTCGGTGGACGAG-3'	This study
Cas9-Cdt1(1/100)Cy(-)-Rv	5'-GCCGCGCGGCCGCGCGGGCCGGTGGCCTGGCCTG-3'	This study
HPRT-Ex3-gRNA-top	5'-CACCGATGTGATGAAGGAGATGGG-3'	This study
HPRT-Ex3-gRNA-btm	5'-AAACCCCATCTCCTTCATCACATC-3'	This study
HPRT-Ex6-gRNA-top	5'-CACCGAGACTTTGCTTTCCTTGGTC-3'	Gravells, P. et al. Hum. Mol. Genet. 24, 7097-7110 (2015)
HPRT-Ex6-gRNA-btm	5'-AAACGACCAAGGAAAGCAAAGTCTC-3'	Gravells, P. et al. Hum. Mol. Genet. 24, 7097-7110 (2015)
SceDR-gRNA-top	5'-CACCGTGTCCGGCTAGGGATAACA-3'	This study
SceDR-gRNA-btm	5'-AAACTGTTATCCCTAGCCGGACAC-3'	This study
SceSA-gRNA-top	5'-CACCGCGCCGAGGTGAAGTTCGATA-3'	This study
SceSA-gRNA-btm	5'-AAACTATCGAACTTCACCTCGGCGC-3'	This study
MSH2-gRNA-top	5'-CACCGAAGAGAAATAATGAATGACT-3'	This study
MSH2-gRNA-btm	5'-AAACAGTCATTCATTATTTCTCTTC-3'	This study
HPRT-5' ext	5'-TTGGAATGTTCACATAGGTGACAC-3'	Saito, S. et al. FEBS J. 284, 2748-2763 (2017)
Universal Primer 2A	5'-CACCGCATGTTAGAAGACTTCCTC-3'	Saito, S. et al. FEBS J. 284, 2748-2763 (2017)
HPRT-3' ext	5'-CTGGCCTATAGCCACCATGGAAGC-3'	Saito, S. et al. FEBS J. 284, 2748-2763 (2017)
Universal Primer D3	5'-CTAGAGGATCATAATCAGCCATACC-3'	Saito, S. et al. FEBS J. 284, 2748-2763 (2017)
SD-Fw	5'-ATCCTGTAATGCTCTCATTGAAACAG-3'	Kurosawa, A. et al. FEBS J. 290, 5313-5321 (2023)
HPRT4	5'-TTGATGTAATCCAGCAGGTCAGC-3'	Kurosawa, A. et al. FEBS J. 290, 5313-5321 (2023)
HPRT-Ex6-5' ext	5'-CCATCCTAAAGGTAAGCCAGGGAG-3'	This study
HPRT-Ex6-3' ext	5'-TGGCTTATATCCAACACTTCGTGG-3'	This study
HPRT-Ex6-N-Fw	5'-CAGACTTTGCTTTCCTTGGTCAGG-3'	This study
HPRT-Ex6-N-Rv	5'-ATCCTCTGCCATGCTATTCAGGAC-3'	This study
RAD52-5' ext	5'-CTCCAGCTATCTTGTTAGCTCCTG-3'	This study
Universal Primer Hyg	5'-CGGTCAATACACTACATGGCGTG-3'	This study
RAD52-Nega	5'-AATGGTCTGTTGGGAGGAGGAACTC-3'	This study
MSH2 GT-Fw	5'-GCCTGTGATCACTAGTGGAGAATGTAGC-3'	Suzuki, T. et al. PLoS One. 8(4), e61189 (2013)
3 LoxP Rv	5'-GAAGAGGTTCACTAGTACTGGCCATTGC-3'	Suzuki, T. et al. PLoS One. 8(4), e61189 (2013)
MSH2-Del-Check-Fw	5'-AGGTAGATCCTTGGTTTGGGCAAC-3'	This study
MSH2-Del-Check-Rv	5'-TATCCTGTTACCAGTGGAGGTGAC-3'	This study
iPCR-HPRT8.9-5-1-Rv	5'-AACAAGTTTTAACACACTGTAGTC-3'	This study
iPCR-HPRT8.9-5-2-Rv	5'-CAGAGTCCCACTATACCACAACTG-3'	This study
iPCR-HPRT8.9-5-3-Rv	5'-ACATCTCGAGCAAGACGTTCAGTC-3'	This study
iPCR-HPRT8.9-5-Fw	5'-CTATGGATCTCGAGCAGCTGAAGC-3'	This study
iPCR-HPRT8.9-3-Rv	5'-GTGGTATGGCTGATTATGATCCTC-3'	This study
iPCR-HPRT8.9-3-1-Fw	5'-GGTCTTCTATAGCCTCCTTCCC-3'	This study
iPCR-HPRT8.9-3-2-Fw	5'-GGGCTATGCAAGGAAGATATACTG-3'	This study
iPCR-HPRT8.9-3-3-Fw	5'-AATCTGGCTGATCCGTACTAATCC-3'	This study
iPCR-HPRT8.9-3-4-Fw	5'-TCTGCCCTGTGATATTCAGAAAGTG-3'	This study
HPRT-F	5'-TGAGGGCAAAGGATGTGTTACGTG-3'	Saito, S. et al. Nat. Commun., 8, 16112 (2017)
HPRT-2.9-Fw	5'-TTTATCTTGTTAGACTACAGTGTG-3'	Saito, S. et al. Nat. Commun., 8, 16112 (2017)
HPRT-3.8-Fw	5'-CACATCACAGGTACCATATCAGTG-3'	Saito, S. et al. Nat. Commun., 8, 16112 (2017)
HPRT-0.7-Rv	5'-TCCACAGTGTCAATGTTGTGATGC-3'	Saito, S. et al. Nat. Commun., 8, 16112 (2017)
HPRT-2.3-Rv	5'-ACAGTATATCTTCCTTGCATAGCC-3'	Saito, S. et al. Nat. Commun., 8, 16112 (2017)
HPRT-5.1-Rv	5'-GGAAGCCCATATTAGAGGATCCAC-3'	Saito, S. et al. Nat. Commun., 8, 16112 (2017)

Supplementary Table 2. Cas9/sgRNA expression vectors used in this study.

Name	Parent plasmid	Oligonucleotides used for vector construction	Target gene	Target sequence*	Use
pX330-Cas9-HPRT-Ex3	pX330-U6-Chimeric_BB-CBh-hSpCas9	HPRT-Ex3-gRNA-top & HPRT-Ex3-gRNA-btm	*HPRT*	5′–GATGTGATGAAGGAGATGGGAGG–3′	Gene-targeting assay / chromosomal DSB joining assay
pX330-Cas9-hGem(1/110)-HPRT-Ex3	pX330-Cas9-hGem(1/110)				
pX330-Cas9-hCdt1(30/120)-HPRT-Ex3	pX330-Cas9-hCdt1(30/120)				
pX330-Cas9-hCdt1(1/100)-HPRT-Ex3	pX330-Cas9-hCdt1(1/100)				
pX330-Cas9-hCdt1(1/100)Cy(-)-HPRT-Ex3	pX330-Cas9-hCdt1(1/100)Cy(-)				
pX330-Cas9-HPRT-Ex6	pX330-U6-Chimeric_BB-CBh-hSpCas9	HPRT-Ex6-gRNA-top & HPRT-Ex6-gRNA-btm	*HPRT*	5′–AGACTTTGCTTTCCTTGGTCAGG–3′	Gene-targeting assay
pX330-Cas9-hGem(1/110)-HPRT-Ex6	pX330-Cas9-hGem(1/110)				
pX330-Cas9-hCdt1(1/100)-HPRT-Ex6	pX330-Cas9-hCdt1(1/100)				
pX330-Cas9-DR	pX330-U6-Chimeric_BB-CBh-hSpCas9	SceDR-gRNA-top & SceDR-gRNA-btm	*SceGFP*	5′–GTGTCCGGCTAGGGATAACAGGG–3′	DR-GFP assay
pX330-Cas9-hGem(1/110)-DR	pX330-Cas9-hGem(1/110)				
pX330-Cas9-hCdt1(30/120)-DR	pX330-Cas9-hCdt1(30/120)				
pX330-Cas9-hCdt1(1/100)-DR	pX330-Cas9-hCdt1(1/100)				
pX330-Cas9-hCdt1(1/100)Cy(-)-DR	pX330-Cas9-hCdt1(1/100)Cy(-)				
pX330-Cas9-SA	pX330-U6-Chimeric_BB-CBh-hSpCas9	SceSA-gRNA-top & SceSA-gRNA-btm	*3'GFP*	5′–CGCCGAGGTGAAGTTCGATAGGG–3′	SA-GFP assay
pX330-Cas9-hGem(1/110)-SA	pX330-Cas9-hGem(1/110)				
pX330-Cas9-hCdt1(30/120)-SA	pX330-Cas9-hCdt1(30/120)				
pX330-Cas9-hCdt1(1/100)-SA	pX330-Cas9-hCdt1(1/100)				
pX330-Cas9-hCdt1(1/100)Cy(-)-SA	pX330-Cas9-hCdt1(1/100)Cy(-)				
pX330-Cas9-MSH2	pX330-U6-Chimeric_BB-CBh-hSpCas9	MSH2-gRNA-top & MSH2-gRNA-btm	*MSH2*	5′–AAGAGAAATAATGAATGACTTGG–3′	*MSH2* knockout

*PAM sequences (NGG) are underlined.

Supplementary Table 3. ***HPRT*** **targeting vectors used in this study.**

Vector	5'-arm			3'-arm			Selection marker	Reference
	Length (kb)	No. of mismatches	Homology (%)	Length (kb)	No. of mismatches	Homology (%)		
p8.9HPRT-2A-Puro	3.8	0	100	5.1	0	100	2A-Puro^R	Saito, S. et al. FEBS J. 284, 2748-2763 (2017)
p8.9HPRT-2A-Puro-95-95	3.8	186	95	5.1	255	95	2A-Puro^R	This study
p8.9HPRT-2A-Bsr	3.8	0	100	5.1	0	100	2A-Bsr	This study
p8.9HPRT-2A-Bsr-95-95	3.8	186	95	5.1	255	95	2A-Bsr	This study
p3.0HPRT-2A-Puro	1.7	0	100	1.3	0	100	2A-Puro^R	This study
p3.0HPRT-2A-Puro-100-99	1.7	0	100	1.3	12	99	2A-Puro^R	This study
p3.0HPRT-2A-Puro-100-97.5	1.7	0	100	1.3	32	97.5	2A-Puro^R	This study
p3.0HPRT-2A-Puro-100-95	1.7	0	100	1.3	65	95	2A-Puro^R	This study
p3.0HPRT-2A-Puro-100-90	1.7	0	100	1.3	131	90	2A-Puro^R	This study
p3.0HPRT-2A-Puro-100-80	1.7	0	100	1.3	263	80	2A-Puro^R	This study
p3.0HPRT-2A-Puro-95-100	1.7	82	95	1.3	0	100	2A-Puro^R	This study
p3.0HPRT-2A-Puro-95-99	1.7	82	95	1.3	12	99	2A-Puro^R	This study
p3.0HPRT-2A-Puro-95-97.5	1.7	82	95	1.3	32	97.5	2A-Puro^R	This study
p3.0HPRT-2A-Puro-95-95	1.7	82	95	1.3	65	95	2A-Puro^R	This study
p3.0HPRT-2A-Puro-95-90	1.7	82	95	1.3	131	90	2A-Puro^R	This study
p3.0HPRT-2A-Puro-95-80	1.7	82	95	1.3	263	80	2A-Puro^R	This study
pHPRT-Ex6-2A-Puro	3.1	0	100	3.0	0	100	2A-Puro^R	This study
pHPRT-Ex6-2A-Puro-95-95	3.1	155	95	3.0	150	95	2A-Puro^R	This study
pHPRT-Ex6-2A-Bsr	3.1	0	100	3.0	0	100	2A-Bsr	This study
pHPRT-Ex6-2A-Bsr-95-95	3.1	155	95	3.0	150	95	2A-Bsr	This study
pHPRT-DR-GFP	1.7	0	100	1.3	0	100	2A-Hyg^R	Morotomi-Yano, K. et al. Sci. Rep. 8, 10344 (2018)
pHPRT-SA-GFP	1.7	0	100	1.3	0	100	2A-Hyg^R	Kurosawa, A. et al. FEBS J. 290, 5313-5321 (2023)

Supplementary Table 4. PCR primers used to amplify the homology arms of *HPRT* targeting vectors.

Name	Sequence	Targeting vector	Arm
HPRT-3.8-Fw	5'-GGGGACAACTTTGTATAGAAAAGTTGCACATCACAGGTACCATATCAGTG-3'	p8.9HPRT-2A-Puro / p8.9HPRT-2A-Bsr	5' arm
HPRT-3.8-Rv	5'-GGGGACTGCTTTTTTGTACAAACTTGCACATCTCGAGCAAGACGTTCAGT-3'		
HPRT-5.1-Fw	5'-GGGGACAGCTTTCTTGTACAAAGTGGATCACATTGTAGCCCTCTGTGTGC-3'		3' arm
HPRT-5.1-Rv	5'-GGGGACAACTTTGTATAATAAAGTTGCTATATTACCCTGTTATCCCTAGCGTAACTGGAAGCCCATATTAGAGGATCCAC-3'		
HPRT-1.7-Fw	5'-GGGGACAACTTTGTATAGAAAAGTTGCAGCAGCTGTTCTGAGTACTTGCT-3'	p3.0HPRT-2A-Puro / pHPRT-DR-GFP / pHPRT-SA-GFP	5' arm
HPRT-1.7-Rv	5'-GGGGACTGCTTTTTTGTACAAACTTGCACATCTCGAGCAAGACGTTCAGT-3'		
HPRT-1.3-Fw	5'-GGGGACAGCTTTCTTGTACAAAGTGGATCACATTGTAGCCCTCTGTGTGC-3'		3' arm
HPRT-1.3-Rv	5'-GGGGACAACTTTGTATAATAAAGTTGTTAATTAATTTAAATGGCCAGTTATACTGCC-3'		
HPRT-Ex6-5-Fw	5'-GAAAAGTTGTCACATCACAGGTACCGGAATGGTATACTTCCTACCTCTC-3'	pHPRT-Ex6-2A-Puro / pHPRT-Ex6-2A-Bsr	5' arm
HPRT-Ex6-5-Rv	5'-CTATACGAACGGTAGCGGCCGCCATTGTTTTGCCAGTGTCAATTATATCC-3'		
HPRT-Ex6-3-Fw	5'-GTAATACGTAACGATGCGATCGCAGTATAATCCAAAGATGGTCAAGG-3'		3' arm
HPRT-Ex6-3-Rv	5'-GAAGCCCATATTAGAGGATCCTACCTGAACTCAGGAATGCTCCAG-3'		

Supplementary Table 5. Antibodies used in this study.

Name	Supplier	Catalogue number	Lot number	Dilution
anti-Msh2	Merck Millipore	NA27	D06571-9	1:1,000
anti-Msh6	BD Bioscience	610918	1085883	1:1,000
anti-Rad52	Abcam	ab124971	-	1:2,000
anti-Rad54	Cell Signaling Technology	15016	1	1:2,000
anti-Rad54B	Gift from Drs. Yasuhara and Miyagawa*	-	-	1:1,000
anti-Lig4	Cell Signaling Technology	14649	1	1:1,000
anti-Polθ	Abcam	ab111218	-	1:2,000
anti-BLM	Abcam	ab476	-	1:1,000
anti-XPF	Santa Cruz Biotechnology	sc-136153	H1721	1:1,000
anti-ERCC1	Santa Cruz Biotechnology	sc-17809	H2316	1:1,000
anti-Cas9	Cell Signaling Technology	14697	8	1:2,000
anti-Cas9	FUJIFILM Wako Pure Chemical	310-08431	01108L	1:2,000
anti-Cdt1	Cell Signaling Technology	8064	2	1:2,000
anti-Geminin	Cell Signaling Technology	52508	1	1:2,000
anti-Topo IIα	BD Bioscience	611326	11964	1:2,000
anti-β-actin	Sigma-Aldrich	A5441	026M4780V	1:5,000
anti-Ku80	BD Bioscience	611360	1313988	1:5,000
anti-mouse IgG	Merck Millipore	AP124P	3032923	1:1,000
anti-rabbit IgG	Merck Millipore	AP187P	3026763	1:1,000

*Yasuhara, T. *et al.* Nat. Commun. 5:5426, 2014.

Supplementary Methods

Construction of *HPRT* targeting vectors harboring mismatches

HPRT targeting vectors harboring mismatches (base substitutions) in the arms were generated by standard molecular biology techniques and artificial gene synthesis (GenScript Japan K.K., Tokyo, Japan). These artificially synthesized arm fragments include: a 1.7-kb (1,684-bp) 5' arm with 95% homology (82 mismatches) to the genome (intron 2), a 1.3-kb (1,323-bp) 3' arm with 99% homology (12 mismatches), 97.5% homology (32 mismatches), 95% homology (65 mismatches), 90% homology (131 mismatches), or 80% homology (263 mismatches) to the genome (exon 2 and intron 3), a 3.8-kb (3,838-bp) 5' arm with 95% homology (186 mismatches) to the genome (intron 1, exon 2, and intron 2), a 5.1-kb (5,103-bp) 3' arm with 95% homology (255 mismatches) to the genome (exon 2 and intron 3), 3.1-kb (3,149-bp) 5' arm with 95% homology (155 mismatches) to the genome (intron 5), and a 3.0-kb (3,005-bp) 3' arm with 95% homology (150 mismatches) to the genome (exon 6 and intron 6). These mismatches were introduced at equal intervals in exonic and intronic regions (other than splice donor/acceptor sites) in a way that does not change the overall GC content of arm sequences (i.e., mismatches were introduced by substituting A for T, T for A, G for C, and C for G). The reference genomic sequence of the *HPRT* gene was retrieved from the Ensembl genome browser (ENSG00000165704.15). Sequences of mutated arms are presented at the bottom of this document.

Generation of Nalm-6 cell lines for GFP reporter assays

The DR-GFP reporter cassette derived from pDRGFP (Addgene plasmid #26475)[23] was inserted at exon 3 of the *HPRT* gene by gene targeting with the pHPRT-DR-GFP vector[66]. This vector was constructed by subcloning a 6.8-kb SspI/BspQI fragment containing the DR-GFP reporter cassette derived from pDRGFP (Addgene plasmid #26475)[23] into the SpeI-digested pENTR lox71-

P 2A-Hyg plasmid[62] containing the 1.7-kb 5' arm and the 1.3-kb 3' arm. pHPRT-SA-GFP was similarly constructed as above using primers HPRT-TV-backbone-Fw and HPRT-TV-backbone-Rv (Supplementary Table 1) and used as a targeting vector to insert the SA-GFP cassette (obtained as synthetic DNA fragments (GenScript Japan K.K)) at exon 3 of the *HPRT* gene. Gene targeting with pHPRT-DR-GFP and pHPRT-SA-GFP was performed in Nalm-6 cell lines using the GTE-1 electroporation apparatus, and transfected cells were doubly selected with 0.4 mg/ml hygromycin B and 20 μM 6TG. The correct insertion of the reporter cassette was analyzed by PCR using primers SD-Fw and HPRT4 (Supplementary Table 1).

Construction of Cas9 expression vectors

Cell cycle-regulated Cas9 expression vectors were constructed using In-Fusion®HD Cloning Kit. Briefly, a 49-mer DNA fragment containing multiple cloning sites was prepared by annealing oligonucleotides Cas9-linker-top and Cas9-linker-btm (Supplementary Table 1). The annealed DNA fragment was ligated with the FseI/EcoRI-digested pX330-U6-Chimeric_BB-CBh-hSpCas9 (Addgene plasmid #42230)[33], yielding pX330-linker, in which the multiple cloning sites including a BamHI site are present at the 3’ end of Cas9 cDNA. The S/G2-Cas9 vector pX330-Cas9-hGeminin(1/110) was constructed using In-Fusion®HD Cloning Kit. Briefly, a fragment containing hGeminin(1/110) cDNA was PCR amplified with PrimeSTAR HS DNA Polymerase (Takara Bio) using primers (Cas9-Geminin-Fw/Cas9-Geminin-Rv (Supplementary Table 1)) and pSce-Cy-G2 [63] as a template, and the PCR fragment was cloned into the BamHI site of pX330-linker. Three G1-Cas9 vectors (pX330-Cas9-hCdt1(30/120), pX330-Cas9-hCdt1(1/100) and pX330-Cas9-hCdt1(1/100)Cy(-)) were similarly constructed using PCR fragments obtained with primers Cas9-Cdt1(30/120)-Fw/Cas9-Cdt1(30/120)-Rv, Cas9-Cdt1(1/100)-Fw/Cas9-Cdt1(1/100)-Rv, Cas9-Cdt1(1/100)-Fw/Cas9-Cdt1(1/100)Cy(-)-Rv or Cas9-Cdt1(1/100)Cy(-)-Fw/Cas9-

Cdt1(1/100)-Rv (Supplementary Table 1) (note that two PCR fragments were used for pX330-Cas9-hCdt1(1/100)Cy(-) construction).

Sense and antisense oligonucleotides of each single-guide RNA targeting sequence were annealed and cloned into the BbsI site of pX330-U6-Chimeric_BB-CBh-hSpCas9, pX330-Cas9-hGeminin(1/110), pX330-Cas9-hCdt1(30/120), pX330-Cas9-hCdt1(1/100), and pX330-Cas9-hCdt1(1/100)Cy(-). The CRISPR-Cas9 target sequences (20-bp target and 3-bp PAM sequence (underlined)) used in this study include: for gene-targeting assay and chromosomal DSB joining assay at exon 3 of the *HPRT* gene, 5′-GATGTGATGAAGGAGATGGGAGG-3′; for gene-targeting assay at exon 6 of the *HPRT* gene, 5′- AGACTTTGCTTTCCTTGGTCAGG-3′; for DR-GFP assay, 5′-GTGTCCGGCTAGGGATAACAGGG-3′; for SA-GFP assay, 5′-CGCCGAGGTGAAGTTCGATAGGG-3′; for *MSH2* knockout, 5′-AAGAGAAATAATGAATGACTTGG-3′.

Junction analysis of RI clones

The p8.9HPRT-2A-Puro or the p8.9HPRT-2A-Puro-95-95 targeting vector was each transfected into Msh2-deficient or proficient $LIG4^{-/-}POLQ^{-/-}$ Nalm-6 cells and the resulting RI clones were subjected to junction analysis based on inverse PCR[3]. Briefly, 10 µg of genomic DNA extracted from RI clones was digested with 30 U of HindIII, BamHI and BclI, or ClaI and BstBI. The digested DNA was precipitated with ethanol and 1 µg of the DNA was self-circularized in 400 µl of ligation buffer at 16°C overnight by using T4 DNA Ligase (Takara Bio). The self-ligated DNA was extracted with phenol:chloroform and then with chloroform alone. Subsequently, the self-ligated DNA was precipitated with ethanol and used as the template for PCR amplification with TaKaRa Ex Premier DNA Polymerase (Takara Bio) or EmeraldAmp PCR Master Mix (Takara Bio). Primers used to amplify 5′-junctions containing DNA were as follows: iPCR-HPRT8.9-5-Fw and iPCR-HPRT8.9-5-1-Rv, iPCR-HPRT8.9-5-Fw and iPCR-

HPRT8.9-5-2-Rv, or iPCR-HPRT8.9-5-Fw and iPCR-HPRT8.9-5-3-Rv. Primers used to amplify 3′-junctions containing DNA were as follows: iPCR-HPRT8.9-3-1-Fw and iPCR-HPRT8.9-3-Rv, iPCR-HPRT8.9-3-2-Fw and iPCR-HPRT8.9-3-Rv, iPCR-HPRT8.9-3-3-Fw and iPCR-HPRT8.9-3-Rv, or iPCR-HPRT8.9-3-4-Fw and iPCR-HPRT8.9-3-Rv (Supplementary Table 1). The PCR products were cloned into pTAKN2 T-Vector to determine the sequence (Eurofins Genomics K.K.). BLAST programs were used to map the junction sequences onto the human genome (University of California Santa Cruz's Genome Browser). Sequence alignments were performed using CLUSTALW programs (Kyoto University Bioinformatics Center).

Chromosomal DSB joining assay

Chromosomal DSB joining assay was performed as described previously[3]. Briefly, pX330-Cas9-hGeminin(1/110)-HPRT-Ex3 or pX330-Cas9-hCdt1(30/120)-HPRT-Ex3 was transfected into Msh2-deficient or proficient *LIG4*$^{-/-}$*POLQ*$^{-/-}$ Nalm-6 cells. After transfection, cells were cultured for 7 days and then selected with 20 μM 6TG. After three weeks of incubation, the resulting drug-resistant (*HPRT*-) colonies were counted, and the mutant frequency was calculated by dividing the number of drug-resistant colonies by the number of surviving cells. Single colonies were isolated, expanded to prepare genomic DNA, and to exclude spontaneous mutants irrelevant to Cas9 cleavage, PCR analysis was performed using primers HPRT-F and HPRT-0.7-Rv. Subsequently, flanking sequences were PCR amplified with primers HPRT-F and HPRT-2.3-Rv, HPRT-2.9-Fw and HPRT 2.3 Rv, HPRT-3.8-Fw and HPRT-2.3-Rv, HPRT-2.9-Fw and HPRT-5.1-Rv, or HPRT-3.8-Fw and HPRT-5.1-Rv (Supplementary Table 1). PCR reactions were performed with EmeraldAmp PCR Master Mix and the PCR products were subcloned into pTAKN2 T-vector to determine the sequence (Eurofins Genomics K.K). Sequence alignments were performed using CLUSTALW programs. Those clones that were picked independently but had identical junction sequences were

regarded as independent clones. DSB joining efficiency was calculated by dividing the number of recombinants by the number of 6TG-resistant clones analyzed. Alu recombination frequency was calculated by multiplying mutant frequency by the DSB joining efficiency.

Sequences of mutated arms of *HPRT* targeting vectors

Red and blue highlight denote substituted bases and exonic sequences, respectively.

Arms for exon 3 targeting vectors

3,838-bp 5' arm with 95% homology to the genome

TCACATCACAGGTACCATATCTGTGTTATTAAATATTTTGTTTGCCAGGGGCTAGACATA
CGAAGACAACCAATATGTGGTACTACTTAAATAATATTAGACTATCTTTTATGATGACAC
TACATGAGTTGACTATAATAAACTTAGACTTCTAAGAGTTTCGGTTTTCAAAAGATCACT
TTGCTTTTTTGGGTGATTTTTGCCCCTTACTGTGAGATGAGTGAGGCTGTTTGGATTTGG
GTTTGGGGTAGCGGGGACAGCTACTTTTCTTTTCTTTTTCTATTTTATTTTGAGGTAGGG
TTTTGCTGTGTCACCCAGGCTCGAGTGCAGTGGTGTGATCTGGGCTCACTGCAACCTCCA
CGTCCCGGGCTCAGGTGATCCACCTGCTTCAGCCTCCCCAGAAACTGGGACTACAGGCGC
GAGCCACATGCCTGGCTAATTATGTATTTTTAGTAGAGATGCGGTTTCACCATGTTGGCC
ACGCTGGTCTCTAACTCCTGAGCTCAGGTGATACGCCCACCAGGGCCTCCCAAAATACTG
GCATTACAGGCATGAGCCGCTCCATCAGCCAGCAGTTTTTCATGTGGTTTTTTTTGTTTG
TATTGTTTTGTTTTGTTTTTGTGATAGGGTCTTACTCTGTTCTCCACGCTGGAGTGCTGT
GCTATGATCGTAGCTCACTGCTGCCTCAAACTCCTGGGCTCTAGTGATTCCTTCTGCCTC
CCCCTCCCGAGTAGCTGGGACAACAGGTATGCACCACCATAGCTGGCAAATTTTTACAAA
GATTTTTGTAGGGACGGGGTCATGCTACATTCCCCATGTCGCTCTTGAACTCCTGGCCTC
ATGCAACTCTCCTGTCTCAGCGTCCCAAAGCACTGGGATTAGAAGTGTGAGCCACCACAC
CTTGCCAGTTTTTCCTGTTCACTGTGATATTTTATCTTGTTTGACTACAGTGTGTTAAAA
CATGTTTTACTAAATTTTCAATCATACTCAAAAGTGGAGAGTATAGTATAATGAATACCC
GAATGTTCATCACCCATGTTTTGAATATTATTAAATATAAACATTTTGCTGCGTTTGTCT
TTGCTCTTTAAAATTTTTCTTATTCTCTTTGTGACCTAAAGCAAATTCCATATCTTATCA
CATTACTTCTACATTCTTGACAAAGATGACTAAGACATATACTTACATGGTTTTTTGTTT
TCTTTTTTGTTTTTTAAAGACCAAATCTCGCTCTTGTCCCCGAGGCTGGAGTGCAATGGT
GGCATCTCAGCTCAGTGCAACGTCTGCCTTCTGGGTACAAGGGATTCTCCTGCCTCAGCC
TGCCAAGTAGCTGGGATTACACGCTCCTGCCACCACGCCTGCCTAATTTTTGTATTTTTA
GAAGAGACGGCGGGGGGAGGTATCACCATGTTGACAAGGCTCGTCTGGAACTCCTGACCT

CTGGTGATCCACCCGCCTCGGGCTCCCAAAGTGCTGGGATTTCAGGCGTGAGCCACCGCG
CGCAGCCTGTTTTTTTGTTTGATTGTTTTGTTTTTTTTGAGTCAGAGTCTTGCTCTGTTT
CGCAGGCTGGAGTGAAGTGGCCCATTCTTGGCTCACTGCAAGCTTCACCTCCCAGGTTCA
ACTGATTCTCCTGCCTCAGCCACCCAAGTAGCTGGGACTACTGGCATGTGTCACCACACC
CCGCTAATTTTTTTGTATTTTAAGTAGAGACGGGATTTCACGGTGTTGCCCAGGCTGGTC
TGGAACTCCTGAGCTCAGGCACTCTGCCTGCCTCAGCCTCCGAAAGTGCTGGGATTACAC
GAGTGAACCAACCCGCCCGGCGTGTTGTTTTCTTACATAATACATTATCATACCTACAAA
GATAACAGTTACTAATATCATGTTACACCTAAATTTCTCTGTTAGACTAAGGTTATTTTT
TTACATCTTAATCCAATCAAATCTTTGTATCCTGTAATGCTCACATTGAAACAGCTATAT
TTCTTTTTCAGATTAGTGATGACGAACCAGGTTATGACCTTGTTTTATTTTGCATACCTA
ATGATTATGCTGAGGATTTGGATAGGGTGTTTATTCCTCATGCACTAATTATGGACAGGT
AAGTAAGATCTTAAAATGAGGTTTTTTACTTTTTCTTGAGTTAATTTCAAACATCAGCTG
CTGTTCTGAGTACTTGCTTTTTGAACATAAACTAGGCCTACTTATTAAATAACTGATGGT
TTCTAAAATCTTCTTTATAAAAAATAAAAGAGGAGGGCGTTACTAATTACTTAGTATCTG
TTGTGGTATAGTGGGACTGTGTAGGGACCAGAACAAAGAAAACATTGAAGGGAGATGGTA
GAAGGAACTCTAGCCAGACTCTTGCATTTCTCAGTCCTTAACAGGGTAATGGACTGGGCC
TGAATCACATGAAGGCAACGTCAGATTTTTATTATTATCCACATCTAGCTTGAAAATTAT
CTGTTAAGTCAATTACAGAGAAAAACCTTACCTGGTATAGAATGCTTGCATTGTATGTGT
GGCTATTCTGTGTTTTTAATTTAAAATTATAATATCAATATATTTGTGTTATAAAATAAT
CTAACTATGGAGGCCATATACAAGAAGACTAAAGTTCTGTCCTTTCAGCCTTCTGTACTC
ATTTCTTCTCAAGCACTGCCTATGCATGTATACTATATCCAAAAGTACATATATACATAT
ATATTTTAACGTATGAGTTTAGTTTTAAATGTTATTGGTCACTTTTAATATTAGTGTGAC
TAGAGCTATCTAATATATATTAAAGGTTGCATAGCATTGTGTCTTATGGAGATACCATAA
CTGATTTAACCAGTCCACAATTGATAGACACTATTTTGATCTTACCGACTGTACTAGATG
AAACATTCTTTTACATGTATGGTACTTGTTCAGCTTTAATCAAGTGGAATTTCTGGGTGA
AGGGGAAAGAGTTTATTGTATATTTTGGTATTGCCAAAATTTCCTCTAAGAAGTTGAAAC
ATTTTATACTCCTGATGTAATATGAGAGTACCTTTCTCATCACAATTTGTCTCTTTTTAT
TTTTTTTTTGAGACAAGGACTCTGTTGCCCAGGCTGGGCTGCAGTGCAGCAGAATGATGA
CAGTTCACTGCAGTCTCATCCTCCTGGGTTCAAGCGATGCTTCCACCTCAGCCTCCTGTG
TAGCTGGGACTATAGGTGAGCGCCACCACTCCCAGCTATTATTTTTATTTTGTAGAAAGA
GGGTTCGCCATGTTACCCTGCCTCCCAAAGTGCTGGGAATACAGGCATGAGCCACTGGGC
CAGTTTCTACAGTCTCTCATAATATTGTATATTATCCATGAAATTTCATTTAATCAGATC
CTGCCAGTCTGATAGGTGTAAATGGTATCTTGTTTTTAATTGCATTTAAAAAAAATTAAG

ATAGTGGTATGCTTGGTTATTTTGAAGGTATCAAATTTATTACCTTATGAAACATGAGCG

CAAAGGATGTGTTACGTGCAAGATTTAAAAAAAAATTTTAAATGCATTTTTTTGAGACATG

GTCTTGCTCTATTGTCCACGCTGGAGTGCAGTGGCACATTCACAGTTCACTCCAGCCTGA

ACATCCTGCACTAAAGTGTTTTTTCCCACCTCACCTCTCTAGTAGCTGGGACTACAGGTTC

ATGCTACCATGCCTGGCTTATTTTTTTTTTTTTTGCAGGGATGGGGTCTCACTATATTGGC

CAGGTTGGTGTGGAAGTTAAATGACTAAGAGGTGTTTGATATAAAGTTTAATGTATGATA

CTTTCTATTAAATTCCTGATTTTATTTCTGTAGGACTGAACGTCTTGCTCGAGATGTG

1,684-bp 5’ arm with 95% homology to the genome

CAGCTGCTGTTCTGAGTACTTGCTTTTTGAACATAAACTAGGCCTACTTATTAAATAACT
GATGGTTTCTAAAATCTTCTTTATAAAAAATAAAAGAGGAGGGCGTTACTAATTACTTAG
TATCTGTTGTGGTATAGTGGACTGTGTAGGGACCAGAACAAAGAAAACATTGAAGGGAG
ATGGTAGAAGGAACTCTAGCCAGACTCTTGCATTTCTCAGTCCTTAACAGGGTAATGGAC
TGGGCCTGAATCACATGAAGGCAACGTCAGATTTTTATTATTATCCACATCTAGCTTGAA
AATTATCTGTTAAGTCAATTACAGAGAAAAACCTTACCTGGTATAGAATGCTTGCATTGT
ATGTGTGGCTATTCTGTGTTTTTAATTTAAAATTATAATATCAATATATTTGTGTTATAA
AATAATCTAACTATGGAGGCCATATACAAGAAGACTAAAGTTCTGTCCTTTCAGCCTTCT
GTACTCATTTCTTCTCAAGCACTGCCTATGCATGTATACTATATCCAAAAGTACATATAT
ACATATATATTTTAACGTATGAGTTTAGTTTTAAATGTTATTGGTCACTTTTAATATTAG
TGTGACTAGAGCTATCTAATATATATTAAAGGTTGCATAGCATTGTGTCTTATGGAGATA
CCATAACTGATTTAACCAGTCCACAATTGATAGACACTATTTTGATCTTACCGACTGTAC
TAGATGAAACATTCTTTTACATGTATGGTACTTGTTCAGCTTTAATCAAGTGGAATTTCT
GGGTGAAGGGGAAAGAGTTTATTGTATATTTTGGTATTGCCAAAATTTCCTCTAAGAAGT
TGAAACATTTTATACTCCTGATGTAATATGAGAGTACCTTTCTCATCACAATTTGTCTCT
TTTTATTTTTTTTTTGAGACAAGGACTCTGTTGCCCAGGCTGGGCTGCAGTGCAGCAGAA
TGATGACAGTTCACTGCAGTCTCATCCTCCTGGGTTCAAGCGATGCTTCCACCTCAGCCT
CCTGTGTAGCTGGGACTATAGGTGAGCGCCACCACTCCCAGCTATTATTTTTATTTTGTA
GAAAGAGGGTTCGCCATGTTACCCTGCCTCCCAAAGTGCTGGGAATACAGGCATGAGCCA
CTGGGCCAGTTTCTACAGTCTCTCATAATATTGTATATTATCCATGAAATTTCATTTAAT
CAGATCCTGCCAGTCTGATAGGTGTAAATGGTATCTTGTTTTTAATTGCATTTAAAAAAA
ATTAAGATAGTGGTATGCTTGGTTATTTTGAAGGTATCAAATTTATTACCTTATGAAACA
TGAGCGCAAAGGATGTGTTACGTGCAAGATTTAAAAAAAATTTTAAATGCATTTTTTTGA
GACATGGTCTTGCTCTATTGTCCACGCTGGAGTGCAGTGGCACATTCACAGTTCACTCCA
GCCTGAACATCCTGCACTAAAGTGTTTTTCCCACCTCACCTCTCTAGTAGCTGGGACTAC
AGGTTCATGCTACCATGCCTGGCTTATTTTTTTTTTTTTGCAGGGATGGGGTCTCACTAT
ATTGGCCAGGTTGGTGTGGAAGTTAAATGACTAAGAGGTGTTTGATATAAAGTTTAATGT
ATGATACTTTCTATTAAATTCCTGATTTTATTTCTGTAGGACTGAACGTCTTGCTCGAGA
TGTG

5,103-bp 3’ arm with 95% homology to the genome

ATCACATTGTAGCCCTCTGAGTGCTCAAGGGGGGCTATATATTCTTTGCTGACCTGCTGC
ATTACATCAAAGCACTGAAAAGAAATAGTGATAGATCCAATCCTATGACTGTAGATTTTT
TCAGACTGAAGAGCTATTGAGTGAGTATATTTAATATATCATTCTTTTTAGTGGCAACAC
TAGGTTTTCTTATATTTTCATTGAATCTCTGCAAACCATTCTTGCTTTCATTTCACTTGC
TTACAGTGAGATTTTTCTATCATATTCACTAGTACTTTAGATCAAAGCCAATACTGTTTA
TTTAAAACTAGTCACCTTGCAGGATATATACTTATTTTAGAGGTGTGTGTGGTTTTTTAT
ATAAACTCCTTTTAGGAATAGCTGTTGGGACTTGGGATAGTTTTTTCACTATACATACTC
GTGACAGATACCCTCTCTTCAGCTACATCGGTTTGTGGGCAGTCAAAAGTCCTTTGGAGG
TAGGTTTGACAAATAAGGTCGGTTAACACTTGTTTCCTACAAAGCACATGGAGAGCTAGT
GTATTGGCGAATTGAAGAATTCCCCCTTTTTTTTTAACAGACTTAAGAAAGGGGACTGCT
GGTATACTCAAGAGAGTAACTCGCACCAGAAACCACTTTAGATCCACAGTCTGCCTGTGA
CACACAATTGAAATGCATCTCAACATTGACACTGTGGATCAAACAAAATCAGTGTGAATA
TTAGTAGTGAATTTCATTCTTAATTTGATCGTGCAAACGATTGATTTTATTACTTTAGT
CTATTGTTTCTGATTTTATCTTGGGTTGGTATTTCCTGTCAGTTACTGTTTTCCTTTAAT
ATAGGAATTTTTCATACTCATCAAAGATTAGAACAAATGACCAGTTTTGCTGTTTCATC
AATGAGTCCTGTCCATCTTAGTAGAAACTCGCCTTATGTACACATTTTTATTGAGAATAT
GACCACTTATCTACATTTATCTATCAACCTCATCCTCTCGATTAATCATCTATTTTAGTC
ACCCAAGTTTTTGACCTTTACCATGTTTACATCAATCCTCTAGGTGATTGGGCAGCCATA
TAAGTATTATTATAGACATATTCACTATCCCATTAAAACGCTTTATGCCCATACATCATT
ACACTACTTCCTACCCATATGCTCCTTTTAACTTGTTAATGTCTTGCTTGAATTAAAGAG
TTGTTTAAACACAAAATTTTGACTTTTACTCAACAAAAGAGATTGATTGATTGATTGATA
GATTGATGGTTTACAGTAGCACTTCATTCTAGTCATTATTGCTGCTGGCAGTATAACTGC
CCAGCCTTTAATACATTGCAGCTTAGAGTCAAAGCATGTTCTTAGAGTTGGTATGATTTT
TCTTTTTGGTCTTCTATAGGCTCCTTCCCCATCCCCATCTGTCTTAATCAGTCTTGTTAG
GTTATGACTAATCTTTGGGCATTTGTGCAGAATGTTATTATAGATAAGCAAAAACGAGCT
AAATAGGGGAGTTTAACTTAAATATTTTCTTTTAAAAAGGATTTCATGTTATAAGATCAT
TTCTGAGTGGTAGAAATGCATTGACATTTTATTTCCATTATCTACTTTTAGTTTTTTTCG
TATTTGTTTAAGATCTTAGTGGATTATTAAGCTGAACTCGTCAACTGATAAAAAGCATGT
CATCTTAAACATAAGCAAACCATATTTTTAGGTTAATTTACACATAGAAAACAGTTTATA
TTATGTGAAATTCTATGTACATATACTATTTTTTTGGTAATTATTGATATGTTTATTTTT
TTTTATTTTATTTTATTTTTTTTTATTTTATTTTATTTAATTATTTTTTTTTTTGAGACT

GAGTCTCACTCTGTTGCCCTGGCTGGAGTGCAGTGGCATCATCGTAGCTCACTGCAACCA
CCACCTCCCGGGTTCAAGCTATTCTTCTGTCTCAGCCTCGCGAGTAGCTGGGACTACAGC
TGCCTGCCACTATGCCCGGGTAATTTTTGTGTTTTTAGTTGAGATGGGGTTTCACCTTGA
TGGTCAGGCTGGTCTCGAAGCCCTGACCTCAGGTGATCCTCCCACCTCAGCCTCCCAAAC
TGCTGGGATTATAGGCATGTGCCACCGTGCCCGGCCGACTTGTTAATTTTTTAAAAAAGC
CTTTACTGGGGTATATTTTTTATAATATAATAATCACATCTTTTAACTATACAATTCCAT
GCTTTTTAGTATATTTATACGGCTATGCAAGGAAGATATTCTGTTAAACAGTAGAAATTC
AGAAAGCTCTTCTGATAATTTCTCTTGATTTGATGATGGGTCATGCCTGTAATCTCAGTC
CTTTGGAAGGCCAAGACAGGAGAATCACTTGAGGCCAGGCGTTCGAGACCAGCCTGGGCT
ACACAGCAATACCCTATCTATACAAATAATAAAAATATCAGTTGATTTGAAGTAAAGTTA
TTTTTTAAAGACAAGGTCTGATTCTGTCACCCAGGCTGGTATGCAGTAGCAAGATCACAC
CTCACTGTGGCCTTGACCTACTGGGCTCAAGTGATTCTCGCACTTCGGCCTCCCGAGTAC
CTGGGACTAACAGGTGTGCTCCACCATGGCTGGCTAATTATTTTTTATGTTTGTAGAGAA
TGGGTCTTACTGTGTTGCCGAGGCTGATCCCGAACTCCTCGGCTCAAGCAGTCTTCCTGG
CTCAGCCTCTAAAATTGCTCGGATTACAGGCTTGAGTCAGCATGCCCAGCCTGAAGTAGG
ATTTCTACCCTGTTTAATATTTCAGCAGCTTGTCATGTATGATATTCATATATGCATATT
AAACATTAGGCAGCTTAATATGGTAAAACTGTAAAATGGTAATTTTAAATTGTTTGCAGG
ATCAATAACATTGATGTCACTATGATTTTTACATGCTGAACTTGACCAATTTGAAACAGA
GAGTTAAAATCTGGCTGATGCGTACTAATCCTAAAGAAAAATTCTATGAACTATTAAATG
TTTCCAGAATATATAAAGATACATTATGATGTCAACACAGCCATCTATTTTTTTTTGGAT
ATAAAAACTCCATTTTTCTAATTAAAGAAAACATGCTTAATAGAAAACATACGGCTGGGA
GCAGTGGCACACATGTAATACCAGTGCTTTGGGAGATCGTGGTGGGAGAATCACTTGAGC
CCAGGAGTTTGAGACCAGCGTAGACAACATAATGAGACCGCCTCTCTACACAAAAAGAAA
TAGTTGTGCATGGTGGCGTCCACCTGTAGTCCCAGCTACATGGGAGGCAGAGGCAGGAGG
ATCCCTTGAGCCTAGGAGTATGAGACTGCAGGAGTTCGACACTGAGTGGAATGCAGTGGT
ACTGCATTCCAGCCTGAGTCACAGAGGGAGACCCTGTCTAAAAAAAATAAGAAAGAAAAG
ACAACTGCAGAAAATTATATAGGATTTAAGTCATTCCAATTATCACTGCCACTTTTTATA
TAGAATATTCTAAAGAATTGTCTCTCTGTGTACACACACTCATATGCGTACTCTTAATCG
AAGTAGCTTGGTAGGATTTAATTTACCTAGTGCCTAGATCGGAAATTGCCTGGGGATTCG
AAATACCTATTTCATTAAAATAAAGATGTCACTGATTTTTAGACTTAACACTATTTTTCT
TACTGCCAAGAAAGAAAACTCTACCAGTTATAAATGTAATTTGCCATCAATTGTAATACT
TCAATTTTAGAGCTATTATAAATAAAATGTGAATGTGCAACTTAGAGCAATGAAATATAC
TACTATATATTTGATGACCATTTCTGCCCTGTGATATTCTGAAAGTGAAAGTTAAATATC

GGCTGAGCATGGTGGCTCAGACCTGTAATCCCAGTACTTAGGGAAGTCAAGACGGGAGGG

TGGCTTGAACCCAGGAGTTGAAGACCAGCCTAGGCAATGAAGCGAGACGCCATCTCAAAT

TATTAAAAATAAGTAAATATGTAAATAAAAAGAAGGTTATGTATACAAATGTATTTCCTA

TGTTGTGAATTTATTTCAAATTTATAGTGATTTTTTTTTATTGAGACGAAGTCTCACTCA

TGTCCCCCAGGCTGGAGTGGGATGGCGTGATCTCAGCTCTCTGCAACCTCTGCCTCCCAC

GTTCAAGCTATACTCCTGCGTTGGCCCCCCGAGTAGCTGCGATTACAGGCGCCTGCTACG

ATGCCTGGCTAATTTTTGTTTTTTTAGTTGAGATGGGGTATCACCATGTTGGCCAGGCTC

GTCTAGAACTCTTGACCTCAGGTGATCCACCCGCCTCGGTCTCCCAAAATGCTGGGATTT

CAGGCGTGAGCCACCGTGCGTGGCCAGTGGTTTTTTGTTCTTGTTGTTGTTGTTGTTTTC

TTTTTGTTTTTGTTTTTGTATTGAGACAGGATCTTGCTCAGTCACCCAGGCTGGAGTGCT

GTGGTGCCATCTTGGTTCAGTGCAACCTCTGCGTGGGCTGAAGCAATCCTCCCACCTCCG

TTTCCAGAGTAGCGGGGACGACAGGTGTGTGCCACCACAGCTGACTAATTTTTGCATTTA

TTTTTGTAGAAACAGGGTTATGCCATGTTGCCCAGGTTGCTCTGAAACTCCTGAGCTCAT

ACAATCCAACTGCCTTGGCATCCCTAAGTGAAATTACAGCCATGGGCCACTGTACCCAGA

CTAGTGATTTTTTTATTTTAATTTTTATTTTATTTTATTATATTTTTTTTACCAAAAAAG

AACAAAGCCTCAGGAGGAATAGTTGATACACAAGTAAATATTATTGGAAATGTTTTTGTC

TGGACCTTAAGCAGAGGGATAATTAGTCTGCATTATGGTCTATCCAGACTAAATGACTGT

TATTAAAATGAAATTATTCATAGGATTTGCAATCTTAGACAAAACTTTTTCATTTTTATA

TTTTTGAGTTACAAATTATGTTCATTTACATTTGAGAACTGTGAGTCACAGAGGGATTAT

GTAACTTACTCAAGATCATTCAAGTCTTTGATTTGAACCGAATCTTTTAACTCTGCAGAT

CTCAGAGTCACTCTTATTTCGAAAAACTTTTTAACTGATCTGGATCCTCTAATATGGGCA

AGG

1,323-bp 3’ arm with 99% homology to the genome

ATCACATTGTAGCCCTCTGTGTGCTCAAGGGGGGCTATAAATTCTTTGCTGACCTGCTGG
ATTACATCAAAGCACTGAATAGAAATAGTGATAGATCCAATCCTATGACTGTAGATTTTA
TCAGACTGAAGAGCTATTGTGTGAGTATATTTAATATATGATTCTTTTTAGTGGCAACAG
TAGGTTTTCTTATATTTTCATTGAATCTCTGCAAACCATACTTGCTTTCATTTCACTTGG
TTACAGTGAGATTTTTCTAACATATTCACTAGTACTTTACATCAAAGCCAATACTGTTTA
TTTAAAACTAGTCACCTTGGAGGATATATACTTATTTTACAGGTGTGTGTGGTTTTTTAA
ATAAACTCCTTTTAGGAATTGCTGTTGGGACTTGGGATAGTTTTTTCACTATACATACTG
GTGACAGATACCCTCTCTTGAGCTACATCGGTTTGTGGGGAGTCAAAAGTCCTTTGGAGC
TAGGTTTGACAAATAAGGTCGGTTAACACTTGTTTCCTAGAAAGCACATGGAGAGCTAGA
GTATTGGCGAATTGAAGAAATCCCCCTTTTTTTTTAACACACTTAAGAAAGGGGACTGCT
GGTATACTCAAGAGAGTAAGTCGCACCAGAAACCACTTTTGATCCACAGTCTGCCTGTGT
CACACAATTGAAATGCATCACAACATTGACACTGTGGATCAAACAAAATCAGTGTGAATT
TTAGTAGTGAATTTCATTCATAATTTGATCGTGCAAACGTTTGATTTTTATTACTTTAGA
CTATTGTTTCTGATTTTATCTTGGGTTGGTATTTCCTGTGAGTTACTGTTTTCCTTTAAA
ATAGGAATTTTTCATACTCTTCAAAGATTAGAACAAATGTCCAGTTTTTGCTGTTTCATC
AATGAGTCCTGTCCATCTTTGTAGAAACTCGCCTTATGTTCACATTTTTATTGAGAATAA
GACCACTTATCTACATTTAACTATCAACCTCATCCTCTCGATTAATCATCTATTTTAGTG
ACCCAAGTTTTTGACCTTTTCCATGTTTACATCAATCCTGTAGGTGATTGGGCAGCCATT
TAAGTATTATTATAGACATAATTCACTATCCCATTAAAACCCTTTATGCCCATACATCATA
ACACTACTTCCTACCCATAAGCTCCTTTTAACTTGTTAAAGTCTTGCTTGAATTAAAGAG
TTGTTTAAACACAAAATTTAGACTTTTACTCAACAAAAGTGATTGATTGATTGATTGATT
GATTGATGGTTTACAGTAGGACTTCATTCTAGTCATTATTGCTGCTGGCAGTATAACTGG
CCA

1,323-bp 3’ arm with 97.5% homology to the genome

ATCACATTGTAGCCCTCTGTGTGCTCAAGGGGGGCTATATATTCTTTGCTGACCTGCTGC
ATTACATCAAAGCACTGAAAAGAAATAGTGATAGATCCATTCCTATGACTGTAGATTTTT
TCAGACTGAAGAGCTATTGTGTGAGTATATTTAATATATCATTCTTTTTAGTGGCAACAG
TAGGTTTTCTTATATTTTCATTGAATCTCTGCAAACCATACTTGCTTTCATTTCACTTGC
TTACAGTGAGATTTTTCTAACATATTCACTAGTACTTTAGATCAAAGCCAATACTGTTTT
TTTAAAACTAGTCACCTTGCAGGATATATACTTATTTTACAGGTGTGTGTGGTTTTTTAT
ATAAACTCCTTTTAGGAATTGCTGTTGGGACTTGGGATAGTTTTTTCACTATACATACTG
GTGACAGATACCCTCTCTTCAGCTACATCGGTTTGTGGGGAGTCAAAAGTCCTTTGGAGG
TAGGTTTGACAAATAAGGTGGGTTAACACTTGTTTCCTACAAAGCACATGGAGAGCTAGA
GTATTGGCGAATTGAAGAATTCCCCCTTTTTTTTTAACACACTTAAGAAAGGGGACTGCT
GGTATACTCAAGAGAGTAAGTCGCACCAGAAACCACTTTAGATCCACAGTCTGCCTGTGT
CACACAATTGAAATGCATCTCAACATTGACACTGTGGATGAAACAAAATCAGTGTGAATA
TTAGTAGTGAATTTCATTCATAATTTGATCGTGCAAACGATTGATTTTTATTACTTTAGA
CTATTGTTTCTGATTTTATCTTGGGTTGGTATTTCCTGTGAGTTACTGTTTTCCTTTAAT
ATAGGAATTTTTCATACTCTTCAAAGATTAGAACAAATGACCAGTTTTTGCTGTTTCATG
AATGAGTCCTGTCCATCTTAGTAGAAACTCGCCTTATGTTCACATTTTTATTGAGAATAT
GACCACTTATCTACATTTAACTATCAACCTCATCCTCTCGATTAATCATCTATTTTAGTG
ACCCAAGTTTTTGACCTTTACCATGTTTACATCAATCCTGTAGGTGATTGGGCAGCCATA
TAAGTATTATTATAGACATTTTCACTATCCCATTAAAACGCTTTATGCCCATACATCATA
ACACTACTTCCTACCCATATGCTCCTTTTAACTTGTTAAAGTCTTGCTTGAATTAAAGAG
TTGTTTAAACACAAAATTTAGACTTTTACTCAACAAAAGAGATTGATTGATTGATTGATT
GATTGATGGTTTACAGTAGCACTTCATTCTAGTCATTATAGCTGCTGGCAGTATAACTGC
CCA

1,323-bp 3' arm with 95% homology to the genome

ATCACATTGTAGCCCTCTGAGTGCTCAAGGGGGGCTATATATTCTTTGCTGACCTGCTGC
ATTACATCAAAGCACTGAAAAGAAATAGTGATAGATCCAATCCTATGACTGTAGATTTTT
TCAGACTGAAGAGCTATTGAGTGAGTATATTTAATATATCATTCTTTTTAGTGGCAACAC
TAGGTTTTCTTATATTTTCATTGAATCTCTGCAAACCATTCTTGCTTTCATTTCACTTGC
TTACAGTGAGATTTTTCTATCATATTCACTAGTACTTTAGATCAAAGCCAATACTGTTTA
TTTAAAACTAGTCACCTTGCAGGATATATACTTATTTTAGAGGTGTGTGTGGTTTTTTAT
ATAAACTCCTTTTAGGAATAGCTGTTGGGACTTGGGATAGTTTTTTCACTATACATACTC
GTGACAGATACCCTCTCTTCAGCTACATCGGTTTGTGGGCAGTCAAAAGTCCTTTGGAGG
TAGGTTTGACAAATAAGGTCGGTTAACACTTGTTTCCTACAAAGCACATGGAGAGCTAGT
GTATTGGCGAATTGAAGAATTCCCCCTTTTTTTTTAACAGACTTAAGAAAGGGGACTGCT
GGTATACTCAAGAGAGTAACTCGCACCAGAAACCACTTTAGATCCACAGTCTGCCTGTGA
CACACAATTGAAATGCATCTCAACATTGACACTGTGGATCAAACAAAATCAGTGTGAATA
TTAGTAGTGAATTTCATTCTTAATTTGATCGTGCAAACGATTGATTTTTATTACTTTAGT
CTATTGTTTCTGATTTTATCTTGGGTTGGTATTTCCTGTCAGTTACTGTTTTCCTTTAAT
ATAGGAATTTTTCATACTCATCAAAGATTAGAACAAATGACCAGTTTTTGCTGTTTCATC
AATGAGTCCTGTCCATCTTAGTAGAAACTCGCCTTATGTACACATTTTTATTGAGAATAT
GACCACTTATCTACATTTATCTATCAACCTCATCCTCTCGATTAATCATCTATTTTAGTC
ACCCAAGTTTTTGACCTTTACCATGTTTACATCAATCCTCTAGGTGATTGGGCAGCCATA
TAAGTATTATTATAGACATATTCACTATCCCATTAAAACGCTTTATGCCCATACATCATT
ACACTACTTCCTACCCATATGCTCCTTTTAACTTGTTAATGTCTTGCTTGAATTAAAGAG
TTGTTTAAACACAAAATTTTGACTTTTACTCAACAAAAGAGATTGATTGATTGATTGATA
GATTGATGGTTTACAGTAGCACTTCATTCTAGTCATTATTGCTGCTGGCAGTATAACTGC
CCA

1,323-bp 3’ arm with 90% homology to the genome

ATCACATTGAAGCCCTCTGAGTGCTCAAGCGGGGCTATATATTCTTTGCAGACCTGCTGC
ATTACATCATAGCACTGAAAAGAAATAGTCATAGATCCAATCCTATGACAGTAGATTTTT
TCAGACTGATGAGCTATTGAGTGAGTATAATTAATATATCATTCTTTTTTGTGGCAACAC
TAGGTTTTCATATATTTTCATTGAATCTCAGCAAACCATTCTTGCTTTCTTTTCACTTGC
TTACAGTGACATTTTTCTATCATATTCACAAGTACTTTAGATCAAAGCCTATACTGTTTA
TTTAAAACTTGTCACCTTGCAGGATATATTCTTATTTTAGAGGTGTGTGAGGTTTTTTAT
ATAAACTCCATTTAGGAATAGCTGTTGGGTCTTGGGATAGTTTTTTCACAATACATACTC
GTGACAGATTCCCTCTCTTCAGCTACATCCGTTTGTGGGCAGTCAAAAGACCTTTGGAGG
TAGGTTTGAGAAATAAGGTCGGTTAACACATGTTTCCTACAAAGCACATCGAGAGCTAGT
GTATTGGCGTATTGAAGAATTCCCCCTTTATTTTTAACAGACTTAAGAATGGGGACTGCT
GGTATACTCTAGAGAGTAACTCGCACCAGTAACCACTTTAGATCCACAGACTGCCTGTGA
CACACAATTCAAATGCATCTCAACATTGAGACTGTGGATCAAACAAAATGAGTGTGAATA
TTAGTAGTGTATTTCATTCTTAATTTGATGGTGCAAACGATTGATTTTTTTTACTTTAGT
CTATTGTTTGTGATTTTATCTTGGGTTGGAATTTCCTGTCAGTTACTGTATTCCTTTAAT
ATAGGAATTATTCATACTCATCAAAGATTTGAACAAATGACCAGTTTTTCCTGTTTCATC
AATGAGTCCAGTCCATCTTAGTAGAAACTGGCCTTATGTACACATTTTTTTTGAGAATAT
GACCACTTAACTACATTTATCTATCAACCACATCCTCTCGATTAATCATGTATTTTAGTC
ACCCAAGTTATTGACCTTTACCATGTTTAGATCAATCCTCTAGGTGATTCGGCAGCCATA
TAAGTATTAATATAGACATATTCACTATCGCATTAAAACGCTTTATGCCGATACATCATT
ACACTACTTGCTACCCATATGCTCCTTTTTACTTGTTAATGTCTTGCTTCAATTAAAGAG
TTGTTTAAAGACAAAATTTTGACTTTTACACAACAAAAGAGATTGATTGTTTGATTGATA
GATTGATGGATTACAGTAGCACTTCATTCAAGTCATTATTGCTGCTGGCTGTATAACTGC
CCA

1,323-bp 3’ arm with 80% homology to the genome

ATCAGATTGAAGCCGTCTGAGTGCACAAGCGGGGGTATATATTCATTGCAGACCAGCTGC
ATTAGATCATAGCAGTGAAAAGAATTAGTCATAGTTCCAATCCTTTGACAGTAGTTTTTT
TCAGTCTGATGAGCAATTGAGTGACTATAATTAAAATATCATTCATTTTTGTGGGAACAC
TAGGATTTCATATAATTTCATTGATTCTCAGCAATCCATTCTTGGTTTCTTTTCTCTTGC
TTACTGTGACATTTATCTATCATAATCACAAGTAGTTTAGATCATAGCCTATACAGTTTA
TTTATAACTTGTCAGCTTGCAGGAAATATTCTTAATTTAGAGGTCTGTGAGGTTATTTAT
ATAATCTCCATTTACGAATAGCTGATGGGTCTTGCGATAGTTTTATCACAATACTTACTC
GTGAGAGATTCCCTGTCTTCAGCTTCATCCGTTTCTGGGCAGTCTAAAGACCTTAGGAGG
TAGGATTGAGAAATTAGGTCGGTTTACACATGTTACCTACAAAGGACATCGAGACCTAGT
GTATAGGCGTATTGTAGAATTCCCGCTTTATTTTAAACAGACTTTAGAATGGGGTCTGCT
GGTAAACTCTAGAGTGTAACTCGCTCCAGTAACCTCTTTAGATCGACAGACTGCGTGTGA
CACAGAATTCAAATCCATCTCAACTTTGAGACTGAGGATCAAACTAAATGAGTGAGAATA
TTAGAAGTGTATTTGATTCTTAATATGATGGTGCTAACGATTGAATTTTTTTACATTAGT
CTATAGTTTGTGATATTATCTTGGCTTGGAATTTGCTGTCAGTTTCTGTATTCCATTAAT
ATAGCAATTATTCAAACTCATCAATGATTTGAACTAATGACCAGATTTTCCTGTATCATC
AATGTGTCCAGTCCTTCTTAGTAGTAACTGGCCTAATGTACACAATTTTTTTGACAATAT
GACCTCTTAACTACTTTTATCTATGAACCACATCGTCTCGATTATTCATGTATTATAGTC
ACCCTAGTTATTGAGCTTTACCATCTTTAGATCATTCCTCTAGGAGATTCGGCACCCATA
TAAGAATTAATATACACATATTCAGTATCGCATTTAAACGCTTTTTGCCGATACTTCATT
ACACAACTTGCTACGCATATGCTCGTTTTTACTTCTTAATGTCTAGCTTCAATTTAAGAG
TTGTATAAAGACAATATTTTGACTATTACACAACTAAAGAGATTCATTGTTTGAATGATA
GATTCATGGATTACTGTAGCACTTGATTCAAGTCTTTATTGCTGGTGGCTGTATTACTGC
CCA

Arms for exon 6 targeting vectors

3,149-bp 5' arm with 95% homology to the genome

GGAATGGAATACTTCCTACCTCTCTTTATTTAGTTTGAAGTATTTTCATTCTAAACATAA
CTCTCTCACTCTATTTATCTATATATATTATATACATATATATCTTAAATTTTATGTATA
TATATATTTATCTTGCTTAGATTTTGTGTTATGTAATATTTGGTACAAAAAAAATAATAT
TTATAATATATAGACTATTTTCCATGTCTTATTATGTGCTAAAGTATATTGTATCTTAGC
ACCGAGACGCTAAGCAGTTTCCTAGGGATACCAGCTAGTAAACTAAGCGAAACCTTTACT
TCCTTTACCTCAGTGGTTCTCAAAATGAGGTTCCCTAGACCAAAAGTTTTAATATCAGAC
AAGAACCAACCGAATCAAAATATCTGTCATGAGGCCCAGCAAGCTATCCTTTAACAAGTT
TCCGAGTCATTCTGATGCATGCTAAGGATTAGGATCCCTTGTTTTTAGTCATAAGTCACT
TTCTCATAAAGGCCTTCCCTGGCCATCGTATATAAAATCTCATGTTTACACACCGTCAAC
TTCGTATACCTCCTCAATACTTTTATTATCCTGATCACTTATCACTATCAGCCTCTCTCT
CTCTCTCACTCTCTCTCTATGTATATAAATATATATATCACTTATCAGTGTCTAACAGCC
TCTCTTTTTATATATATAATCTATAGAATATATATATATGCAGCATTCTGCAATCATTAT
CACGCTCTATTTTAAAACATTTTCATTCCCCCACAAAGAAACCCAATGCCCTTAGCCATC
ACTCCCCTTTTCCCCTTCCCCCAGCACGTAGCAAACTGATCATCTACGTACTTGCTGTCT
ATAAGATATGCCTATTCTGGACATTTTCTATAAATAGAATCATACAAAATGTGGCCTTTT
GTATCTGCCTTCTCTCACTTAATGTTTACAAGGTTCATTCATGTTGTCGAGTATATCTGC
ACTCATTACCTTTTTATTGCCAAATTGAATGGATAGACAGGTGTTCCACAACTGTGTCCT
GATAAACGCATCTGAAGTTGAAAATATGATAAGTTGAAAATGGATTTTCTACTTTGATAA
ATCTATCGTAAAGTCAGAAAAATCTCAAGTTGAACCATCGTAAGTTGCATACCATCTGAA
TTACATTATTGTTATCCATTCACTGGTAGACAGACGTTAGGTTGTTTGCACTGTTTGCTC
CTTATTTGTCGTACCTGAAATGTCCTTTTTCCCTCCCTTCTTATCCCTTGTTTAAGTCAT
TTAAGACGCAGCTCAAACGTCACCTCCTCAAAACCTTCCTTGATACCGCTTTCCTCTTCA
ATTCACTAGGACCTTTTGCATTTAATTATAATTTTTATTTTTTTTAACACAGAGTCTCAC
TCTGTCAGCAGGCTGGAGTGCAGTGGTTTGATCTCAGCTCACTAACTTCTCTGCCTCCCA
GGTTCAACCAATTCTCATGTCTCAGCCACCCAAGTAGCTGGGACTACTGGTGTGCGCCAC
CATGCCTCGCTAATTGTGTGTGTGTGTCTGTGTATGTATGTATGTATTTATGTGTGTGTG
TGTATATTTATATATACACACACATATTTAAATATATATACATATATTTATATACACACA
TATATAATTATATATACATATATATATTTACACACACACACACATATTTATATATATAGT

TTTTTTTATTTTTAAGTAGAGATGGGGATTTGCCATGTTGGCCAGGCAGGTCTGGCCTCA

AGCCATCGTCCCACCTCGGCCTCGCAATGTGCTGGTATTATAGGCATCAGCCACTGTGCC

TGGCCTGGATTTCATTTTAATTATAAATTATTTTGAACTCAGAAAAATGGGTATGCTGAA

TACCTACCTACCCACAAAAGTATTAACTTTTTGCCATATTTGCTTCTCATCTTATTTTTT

TTGAGAATTTAAAGATCATAATACAACAAAAGCCCCATTTCTTTCCCATCATTCCCAGAA

GTATGACTATTATCCTTAAAGTTGATAAATATCATTCCCATGCATGTATTTTATACTTCC

CTAGTACTAGTTAGCTGTATCCTCTGCACAGGGGCTCATCAAGCTGATTCAAGGGACTCA

TGATCCTGTTCAAAGTTCCTTCAGGTTCTTGGCAGAATTTAGTTCCTAGTGATTGTAGGA

CTGAGGGGCCGTTTTCTCACTGGCTGCAGGCCAGGGGTTGCTCCCAGTTATTTAAAGGCT

CATGCCCAAGCCCATGACAGTCTCACATCATGGCAGCTGACTTCTTCTAAACCAGCAGGA

GAATCTTCCTCTAGTCTACCACATAACGTAATCACAGGAGCGGCTATGCCGTTATTTTCA

CAGATCCAGGTCACATTCAAGGGGAGGCAACCCTTCTGTGTGTGTACTCCAGGAGGCAGG

AATTTTTATTTTCTTTTTCTTTTTTGTAAAAAAGTCTTAAAGTCTTTAATCCCTAAAGGA

GGCAGGATTTTTGAGAGCCATCAGAATACTGCCTACCACAGCCCAGATATCTGCATTTTT

CACAAGTGTCCAGCCATGATGTTTCTGTTGGCTCACACTGCTTTATTGCATTTTTAAAGA

GTATTTTAATTGAAAAGCATTAGGGTTTTGGTTTAAAAAATATTTTCGCTAACAAAGATG

GGTTTGTATAGAGTCCTACTTTTGACTTAATAGCTGAGATTCACTTTAATGTAAAGTCAT

TTTATAGGGTTATTAATTTGGGTGCCTATAAAAATAGTATAAAGCATCTTTCTCGAGTGT

AGTCTGTAAGCCACCTATATTGGAGAGATGGGAGGAGAGAGTCTCTAACTTGAATTTATG

GGAAAAAATCTAAAATACTTTTTATAAAGAAGGACAACATCATAACTGCCTAATAAAATG

TGCATGTTTATATTCAAATTTGCTGTCTTTGATCCTGCACCTACAAATTCCAGTCCTGGG

GGCTGGCTTTCTTACTGCTTGCTGAGGCCCAGATGATATAGATTCCACAATATCTCCATG

TAGATTTAGGTGAGAATTACTGTGCTGTAAAGAATGACAGTATTGCACTTATACATGGGG

GTTTTGGAACTTTATATTGTGACTCTGTATTTAAAGCTATGCAATGTCTTCTTTTTTGAA

AGGATATAATTGACACTGGCAAAACAATG

3,005-bp 3’ arm with 95% homology to the genome

GCAGTATAATCCAAAGATGCTCAAGGTCGCAAGGTATGTTTGACATTTTGACACAGAATT
TTTTCCTCATTTGAAGGGGCATTAAGTGATTGCTTCTTTTTAAGGATAAATGTTTTCAAG
TGTCATTTTATCTTCGAAATGTAATGTAATCTCATATAACACTTAAGATATAATCCTTTA
AAATAATTTTGTCATGTGTAAATAAAGCTCATAATTACACTCACTTCCTTGCCTAATATA
AACATTTGGTTTTTCAGCAAGCTAATTATATCAGTTTGTGCTGAATAGCATGGCAGAGGT
TTTTGGGCCCCCTTGCAAATTTAAGAATAAGGATTCCAATGCGGGTGAGGAAGTGATAGC
AAGGGGTGGGCCCTGAAGAACTGGACCTCCTGGAATTGACTGATGAATGCTGCATCTTCA
TTGTGTCTGTAGTGAAATTATATAATGCCTGCTTCCTTTATTATTAAGTCGGCCTCACCA
CCTCACCTTACCTATGCTGATTTACTTTTGCTTTTATAGATCTACCTGTGTTTATTTCTG
ATTTTCGTTTCATCTCTCATCAACTCTGGGGTGGCATTAATATTCCCACTTTTCAGATAT
GGTTACTGAGGCATAGGGATTTGTCCAAAGGTACAGAGCAAGTCCGCTATAGAGATGAGT
TTTGAACCCAGGGAACCTGCCTCACAGTTTATGCTTTTGGTACCTTAAGTTTTTAATAGT
GTGACATCAAACAAACATTAAAGAATATGTTTTTCTTTTGCTTTTATAATTTCATTAAAT
ACATTAAGTCTCTGATCAGACTGCAGTTTTTATGTAGGGCTCAGGTAATGTTCTAACTTG
TGCTTTTTCCTAAGTGATTTACAGGTTTTTATAAGCCCTATTGAAAAAATCACGGTATCA
GTCGAGCATCTTTGAATCACAGTAAGCCTTCTAGTGAGTGATATGTCAGCAGTTTGACTC
TATGGGCTTTTCTAATATCGAGTTCAAGTGTTTATCAGTCAGTTTTTCTTTTAAATAGAA
TTGGGACAGGTACTATGAGTGTATATAAGTGATACGTTAAAGGACACTAACTAGTATCCA
ATGAAATGGCAAAAACTGCTATCACTTTTGCACCAACCATATAGAAACTAATCAGTGCAG
TTGCTTATTTTTCTACATGGTCTTTAGGGTTTTAAATGTGAACCTACTGTGGCATAGACA
TTAATCCTCTGGGTATTCTATTGTTGTTCTTTCCTGGTAAATGCTGTGGAATTGAGATAC
ACTGGTTCGTGAGCGAGAGTTTTTGTGTTGCCACAGGTACGACATGCTCAAACAATACTA
GGGTCATTTCTTGACCCAACTCATCTATTCACCATAGTTATGTAGCACCGATCTTGCATT
CATTTCATGTATCTTCTTTCAACCCCACGTCAGTGCTGCATATATGATACTCAGAAATTT
AACACTAAGGAATAAGATTATCAGGTAGGATTGAGTTTTCGAGGGTCACAAATCTTGTAT
TGTCTAATATTTCCACTCTGCCTGCTGAGAATTAGTTTTCGCTTCCTTGGAGGTGATATG
GCCTCTGTTGAGTATAAGTCGCCTACTGTGATCACACCAGTGCACTCCAGCCTGGGTGAG
AGAGTGAGACCCTGTCTCACAAAAAAAAAAAAAAAAAAACAATGCATGGCCTAGATGACA
TCTAAGGTTTTTCCCACCCTGTTCCAGTTTTCATGTTCTTGGCAGAGCAGTAAAGTGAGT
AACACATGGACTTGGGAGTAATAGTCTCGCATTTCACTGCGACTTAATCTGAGCGACTATA
CCATATTTAATCTCTCTGATTGTATTTACTCATCTTTAATGGGGAATGATTATTAACATG

TTTTTCTCAGGGAAACTATTTGAGTCAAGGAGATAATATTTTTGAAAATCTTTTTAACTC
CAAAGCGCTGTTTCACTGTTGGTTATAATGTGATTGATCACATTGTAGTGAGCAGCTGCA
TAATTGCGTTTTAGAATGTTGGGAAGATAGTAATATTTTACACATTATATATGTAGCTGC
TTCTGGAACTGTAAACATAGTCCTTTTTTATGGAGATCTCAGTCACGTACCATAAAATTG
ACTCTTTTAAAGTTGTACATTCCAGTGGTTTTTGATATAATCAGAGTTGTGCATCTGCTT
CCACTATTTCATTTTGGAAGCCAAAGAAACCTTGTACCCTTTAGCAGTCATTCTCCCTTG
TCCCAGCCCCTGGCAACTAGTAATCTACTTTCTACAGAATGTCCGTACAGATTTGTGTAA
TATGGACATTCCATATAAAAGGACTCATGCAATATCCTGACTTCTTTCACTTAGCATAGA
GTTTTCAAGGTTCATCTAGCTTGGGGCATGTATCAGTACATCATCCCTTGTTTTGGCTGT
ATAATATTTCATTGTACAATTATATCACATTTTGCTTATGCATCTGTTGGTGAACATTTC
AGTTTCTACCTGTTGGCTTATATGAATAATGTTGATTTGTATGTTTGTGTACAAGTATGT
ATACCTGTTTTCAGGTCTCATGAGTATATAGTTGCTAGGACATATAGTAACTCTGTGTTA
AACATTTTGAGGAATTGCCGGACTATTTAACAAGGTATAAGTACTGTTTTACACCAGTAT
CATATGAGGGTTCCAATATGTCCACATCCTTGACAACACATGTTACTGTCCTTTTTATTC
TAGCCATCCTAGTGGCTATCATGTGGTATCTCATTGTGGATTTGATTTGTGTTTCTCTGT
TGCTGATGATGTTGAACATCTTTTCATCTGCTTATTGGCGATTTACATATATCTTCTTAT
GAACGGTTACCCATTTACACTATGGAAAATGCTTCAGATCCAACTCTAGTCATGCCTTAC
AGATGGAGCTTTATTAAACTTTCAGATCTCTAGGCATATCAAGTGCTGAGTTCTCTTGAT
CTCCTAATACAGATTGCACAGAGTTTAGTGATACCTTTTGTGGAGCATTCCTGAGTTCAC
GTAGG
