# Supplementary material for: Characterization and regulation of cell cycle-independent noncanonical gene targeting
Source: Nat Commun. 2024 Jun 18;15:5044. doi: 10.1038/s41467-024-49385-9 (PMC11189520; doi:10.1038/s41467-024-49385-9)
Supplement: Supplementary file 3 — Reporting Summary [file 41467_2024_49385_MOESM3_ESM.pdf]

nature portfolio

Corresponding author(s): Noritaka Adachi

Last updated by author(s): May 15, 2024

Reporting Summary

Nature Portfolio wishes to improve the reproducibility of the work that we publish. This form provides structure for consistency and transparency in reporting. For further information on Nature Portfolio policies, see our Editorial Policies and the Editorial Policy Checklist.

Statistics

For all statistical analyses, confirm that the following items are present in the figure legend, table legend, main text, or Methods section.

n/a	Confirmed	
☐	☒	The exact sample size (*n*) for each experimental group/condition, given as a discrete number and unit of measurement
☐	☒	A statement on whether measurements were taken from distinct samples or whether the same sample was measured repeatedly
☐	☒	The statistical test(s) used AND whether they are one- or two-sided<br>*Only common tests should be described solely by name; describe more complex techniques in the Methods section.*
☒	☐	A description of all covariates tested
☒	☐	A description of any assumptions or corrections, such as tests of normality and adjustment for multiple comparisons
☐	☒	A full description of the statistical parameters including central tendency (e.g. means) or other basic estimates (e.g. regression coefficient) AND variation (e.g. standard deviation) or associated estimates of uncertainty (e.g. confidence intervals)
☐	☒	For null hypothesis testing, the test statistic (e.g. *F*, *t*, *r*) with confidence intervals, effect sizes, degrees of freedom and *P* value noted<br>*Give P values as exact values whenever suitable.*
☒	☐	For Bayesian analysis, information on the choice of priors and Markov chain Monte Carlo settings
☒	☐	For hierarchical and complex designs, identification of the appropriate level for tests and full reporting of outcomes
☒	☐	Estimates of effect sizes (e.g. Cohen's *d*, Pearson's *r*), indicating how they were calculated

Our web collection on statistics for biologists contains articles on many of the points above.

Software and code

Policy information about availability of computer code

Data collection	No software was used for data collection.
Data analysis	Data analysis was performed using GraphPad Prism (version 8.4.3) and Microsoft Excel (version 16.16.11).

For manuscripts utilizing custom algorithms or software that are central to the research but not yet described in published literature, software must be made available to editors and reviewers. We strongly encourage code deposition in a community repository (e.g. GitHub). See the Nature Portfolio guidelines for submitting code & software for further information.

Data

Policy information about availability of data

All manuscripts must include a data availability statement. This statement should provide the following information, where applicable:

- Accession codes, unique identifiers, or web links for publicly available datasets
- A description of any restrictions on data availability
- For clinical datasets or third party data, please ensure that the statement adheres to our policy

All data generated in the study are included in this published article including supplementary figures, or are available from the corresponding author upon reasonable request.

Research involving human participants, their data, or biological material

Policy information about studies with human participants or human data. See also policy information about sex, gender (identity/presentation), and sexual orientation and race, ethnicity and racism.

Reporting on sex and gender	N/A
Reporting on race, ethnicity, or other socially relevant groupings	N/A
Population characteristics	N/A
Recruitment	N/A
Ethics oversight	N/A

Note that full information on the approval of the study protocol must also be provided in the manuscript.

Field-specific reporting

Please select the one below that is the best fit for your research. If you are not sure, read the appropriate sections before making your selection.

☒ Life sciences ☐ Behavioural & social sciences ☐ Ecological, evolutionary & environmental sciences

For a reference copy of the document with all sections, see nature.com/documents/nr-reporting-summary-flat.pdf

Life sciences study design

All studies must disclose on these points even when the disclosure is negative.

Sample size	Sample sizes were based on preliminary data and published studies, to identify statistically significant changes in each relevant assay.
Data exclusions	No data was excluded from the study.
Replication	All experiments were independently replicated, as described in each figure legend.
Randomization	N/A
Blinding	N/A

Reporting for specific materials, systems and methods

We require information from authors about some types of materials, experimental systems and methods used in many studies. Here, indicate whether each material, system or method listed is relevant to your study. If you are not sure if a list item applies to your research, read the appropriate section before selecting a response.

Materials & experimental systems

n/a	Involved in the study	
☐	☒	Antibodies
☐	☒	Eukaryotic cell lines
☒	☐	Palaeontology and archaeology
☒	☐	Animals and other organisms
☒	☐	Clinical data
☒	☐	Dual use research of concern
☒	☐	Plants

Methods

n/a	Involved in the study	
☒	☐	ChIP-seq
☐	☒	Flow cytometry
☒	☐	MRI-based neuroimaging

Antibodies

Antibodies used	anti-Msh2 antibody (1:1,000, catalogue #NA27, lot #D06571-9, Merck Millipore)<br>anti-Msh6 antibody (1:1,000, catalogue #610918, lot #1085883, BD Bioscience)<br>anti-Rad52 antibody (1:2,000, catalogue #ab124971, Abcam)<br>anti-Rad54 antibody (1:2,000, catalogue #15016, lot #1, Cell Signaling Technology),

anti-Rad54B antibody (1:1,000)
anti-Lig4 antibody (1:1,000, catalogue #14649, lot #1, Cell Signaling Technology)
anti-Polθ antibody (1:2,000, catalogue #ab111218, Abcam)
anti-BLM antibody (1:1,000, catalogue #ab476, Abcam)
anti-XPF antibody (1:1,000, catalogue #sc-136153, lot #H1721, Santa Cruz Biotechnology)
anti-ERCC1 antibody (1:1,000, catalogue #sc-17809, lot #H2316, Santa Cruz Biotechnology)
anti-Cas9 antibody (1:2,000; catalogue #14697, lot #8, Cell Signaling Technology or catalogue #310-08431, lot #01108L, FUJIFILM Wako Pure Chemical)
anti-Cdt1 antibody (1:2,000, catalogue #8064, lot #2, Cell Signaling Technology)
anti-Geminin antibody (1:2,000, catalogue #52508, lot #1, Cell Signaling Technology)
anti-Topo IIα antibody (1:2,000, catalogue #611326, lot #11964, BD Bioscience)
anti-β-actin antibody (1:5,000, catalogue #A5441, lot #026M4780V, Sigma-Aldrich)
anti-Ku80 antibody (1:5,000, catalogue #611360, lot #1313988, BD Bioscience)

Validation

Anti-Msh2, anti-Msh6, anti-Rad52, anti-Rad54, anti-Rad54B, anti-Lig4, anti-Polθ, anti-BLM, anti-XPF, anti-ERCC1, anti-Topo IIα, and anti-Ku80 antibodies: validated by using gene knockout or gene knockdown (this study, PMID:18596031 and PMID:16325483). Other antibodies listed above were purchased commercially and validated by suppliers for their performance.

Eukaryotic cell lines

Policy information about cell lines and Sex and Gender in Research

Cell line source(s)

Nalm-6 was provided by Dr. Koyama as wild-type S14, which is available from Horizon Discovery Group plc (Cambridge, UK). Msh2-deficient mutant cell lines from Nalm-6 (LIG4/POLQ, RAD54/RAD54B, and BLM knockouts; all previously published) were from Adachi lab stocks. Rad52 knockout cell lines were generated by conventional gene targeting without using CRISPR/Cas9.Msh2-proficient cell lines were generated from those mutants by restoring MSH2 expression using a standard gene-targeting method without CRISPR/Cas9 technology. HT1080 was obtained from Institution for Fermentation (Osaka, Japan). A Msh2-deficient HT1080 cell line was generated using CRISPR/Cas9 technology. MDA-MB-436 was obtained from American Type Culture Collection (HTB-130, ATCC, Manassas, VA, USA).

Authentication

Nalm-6 and HT1080 cell lines were both authenticated by short tandem repeat analysis.

Mycoplasma contamination

All cell lines were confirmed negative for mycoplasma contamination.

Commonly misidentified lines
(See ICLAC register)

All cell lines used in this study are not listed in the database of commonly misidentified cell lines maintained by ICLAC.

Plants

Seed stocks

N/A

Novel plant genotypes

N/A

Authentication

N/A

Flow Cytometry

Plots

Confirm that:

☒ The axis labels state the marker and fluorochrome used (e.g. CD4-FITC).

☒ The axis scales are clearly visible. Include numbers along axes only for bottom left plot of group (a 'group' is an analysis of identical markers).

☒ All plots are contour plots with outliers or pseudocolor plots.

☒ A numerical value for number of cells or percentage (with statistics) is provided.

Methodology

Sample preparation

HT1080 cells were transfected with Cas9 expression vectors. After a 48-hr incubation, cells were incubated for 1 hr in growth medium containing 5 µg/ml Hoechst 33342 (DOJINDO Laboratories, Kumamoto, JAPAN), detached with trypsin, and re-suspended in growth medium containing 5 µg/ml Hoechst 33342.

Instrument

Cell Sorter SH800S (Sony Corporation, Tokyo, JAPAN)

Software	Sony Cell Sorter Software (version 2.1)
Cell population abundance	Approximately 1,000,000 cells were collected per G1 and S/G2/M phase.
Gating strategy	Cells were gated according to forward scatter (FSC) and backscatter (BSC) parameters, and sorted according to their DNA content. Specifically, isolated G1 cells correspond to the area shown in magenta (note that early S-phase cell contamination is thus avoided), while S/G2/M cells correspond to the area shown in light blue (Supplementary Fig. 9c).

☒ Tick this box to confirm that a figure exemplifying the gating strategy is provided in the Supplementary Information.
